# Neuroscience of Object Relations in Health and Disorder: A Proposal for an Integrative Model

**DOI:** 10.3389/fpsyg.2021.583743

**Published:** 2021-03-15

**Authors:** Dragan M. Svrakic, Charles F. Zorumski

**Affiliations:** ^1^Department of Psychiatry, Washington University School of Medicine in St. Louis, St. Louis, MO, United States; ^2^Department of Psychiatry, Taylor Family Institute for Innovative Psychiatric Research, Washington University School of Medicine in St. Louis, St. Louis, MO, United States

**Keywords:** object relations, molecular- and systems-neuroscience, trans-disciplinary integration, emotion-enhanced episodic memory, systems-consolidation of memory, cognitive neuroscience

## Abstract

Recent advances in the neuroscience of episodic memory provide a framework to integrate object relations theory, a psychoanalytic model of mind development, with potential neural mechanisms. Object relations are primordial cognitive-affective units of the mind derived from survival- and safety-level experiences with caretakers during phase-sensitive periods of infancy and toddlerhood. Because these are learning experiences, their neural substrate likely involves memory, here affect-enhanced episodic memory. Inaugural object relations are encoded by the hippocampus-amygdala synaptic plasticity, and systems-consolidated by medial prefrontal cortex (mPFC). Self- and object-mental representations, extracted from these early experiences, are at first dichotomized by contradictory affects evoked by frustrating and rewarding interactions (“partial object relations”). Such affective dichotomization appears to be genetically hardwired the amygdala. Intrinsic propensity of mPFC to form schematic frameworks for episodic memories may pilot non-conscious integration of dichotomized mental representations in neonates and infants. With the emergence of working memory in toddlers, an activated self- and object-representation of a particular valence can be juxtaposed with its memorized opposites creating a balanced cognitive-affective frame (conscious “integration of object relations”). Specific events of object relations are forgotten but nevertheless profoundly influence the mental future of the individual, acting (i) as implicit schema-affect templates that regulate attentional priorities, relevance, and preferential assimilation of new information based on past experience, and (ii) as basic units of experience that are, under normal circumstances, integrated as attractors or “focal points” for interactive self-organization of functional brain networks that underlie the mind. A failure to achieve integrated object relations is predictive of poor adult emotional and social outcomes, including personality disorder. Cognitive, cellular-, and systems-neuroscience of episodic memory appear to support key postulates of object relations theory and help elucidate neural mechanisms of psychodynamic psychotherapy. Derived through the dual prism of psychoanalysis and neuroscience, the gained insights may offer new directions to enhance mental health and improve treatment of multiple forms of psychopathology.

## Introduction

For decades, the sole empirical support for object relations theory, a psychoanalytic metapsychology about the development of the human mind, was the effectiveness of psychodynamic psychotherapy, derived from this theory, in the treatment of pathological object relations in personality disorder. Here, our main hypotheses are that (i) cellular- and systems-neuroscience provide insights that may help integrate object relations theory and neural mechanisms of systems-consolidation and semanticization of affect-enhanced episodic memories derived from early interactive experiences with caretakers, and (ii) cognitive neuroscience provides insights that may explain the emergence of the sense of self from engramized autobiographical memories. Although mechanistic studies have been done mainly in rodents, and this is a significant limitation, the processes being studied involve homologous brain systems and fundamental neural functions that are shared with humans, making them potentially informative for understanding human emotion and episodic memory. Studies of contextual fear conditioning in rodents, a form of affect-enhanced episodic-like memory (Eichenbaum, 2017; Tonegawa et al., 2018), combine recombinant DNA and optogenetics to delineate a cascade of cellular- and system-level neural events involved in encoding and long-term consolidation of episodic memories (Kitamura et al., 2017). In humans, functional magnetic resonance imaging (fMRI) with multivariate pattern-analyses (van Kesteren et al., 2016) or electrophysiological recordings (Siapas et al., 2005) are used to study distributed cortical and subcortical patterns of brain activity involved in generalization of episodic memories into associated knowledge structures or schemas. The plastic hippocampus-amygdala-mPFC circuitry is currently best described but future studies will likely add other circuits to the neuroscience of object relations and, generally, the organizational infrastructure of the human mind. Beck's cognitive theory (Beck, 2011) is consistent with modern neuroscience of the formation and self-reinforcing expansion of schematic frameworks of episodic memories (Tse et al., 2011; van Kesteren et al., 2016). Here, we integrate Beck's concept of core beliefs into the continuum of widening cognitive-affective templates for interpreting new experience, piloted by object relations and finalized into neocortical schemas. The templates are characterized by initially strong amygdala involvement, with time increasingly framed by cognitive processing, but are never purely emotional or purely rational. Beck's cognitive model, however, does not account for the preconceptual psychopathology of partial (unintegrated) object relations of personality disorder. We also draw from the higher order theory of emotional consciousness (HOTEC) (LeDoux and Brown, 2017) to account for mental differentiation between one's own self and the object world, a major milestone in mind development. Trans-disciplinary integrations are heuristic models that involve at least some informed speculation, which can be tested through epistemic iterations as knowledge and technology advance. The transdisciplinary model presented here offers testable directions in research to (i) enhance healthy plasticity in critical neurocircuits to promote well-being, (ii) activate this plasticity in psychotherapy, and (iii) capitalize on enhanced plasticity in the developing human brain for early correction and possibly prevention of multiple forms of psychopathology.

### Object Relations Theory: The Basics

Klein (1935, 1946) observed that infants manifest an immediate deep bond with caretakers, usually the mother, and that these early interactions mediate and modulate mind development. Klein's theorizing challenged Freud's “drive/structure” model (Greenberg and Mitchell, 1983), whereby innate sexual- and aggressive drives mediate the development of intrapsychic structures^1^. This challenge marked the birth of object relations theory, a departure from classical psychoanalysis albeit still rooted in its main postulates, the dual-drive theory and the tripartite, Id, Ego, and Superego structure of the mind. Over the years, the theory has evolved into its present form. Fairbairn (1952) considered the innate need for emotional attachment to an object (“object relatedness”), to be the driving force behind mind organization, rather than pleasure-seeking as the goal to itself. This is known as the “relational/structure model” (Greenberg and Mitchell, 1983)^2^, whereby “the interpersonal and the intrapsychic realms create….each other” (Mitchell, 1988, p. 9), a major departure from Freudian psychoanalysis. Ego-psychologists, while still holding that libido and aggression drive the differentiation of the mind, make several major contributions to object relations theory. Hartmann (1950) and Jacobson (1964) explain the formation of self-representations in the mind, hitherto unaccounted for by Klein, who uses the terms “ego” and “self” interchangeably. Hartmann (1950) and Jacobson (1964) also revise Freud's dual-drive theory, which paves the way to integrate unconditioned affects (as defined by Panksepp, 2011) in mind development. Finally, Hartmann (1950), Fairbairn (1952), and Jacobson (1964) re-conceptualize object relations insofar that what the infant internalizes is not an *image* of the object, a position long held by Mitchell (1981), but rather the *interactive experience* with the object. This revision provides the framework for understanding object relations through the prism of episodic memory, the main focus of this work. Kernberg (1975) further advances the integration of ego-psychology, affect theory, and object relations theory, revolutionizes the concept of personality disorder (Kernberg, 1975), and pioneers work on the neurobiological correlates of object relations (Kernberg, 2014).

An object relation is defined as a primordial cognitive-affective unit of the mind derived from early interactive experiences with significant objects, usually the mother^3^, during phase-sensitive periods of infancy and toddlerhood. An object relation consists of three components: a self-representation, an object-representation, and a representation of an affectively charged interactive experience (Auchincloss and Samberg, 2012). The affective valence of the interaction is modulated by the neonate's heritable affect-reactivity and/or quality of caretaking. The most influential object relations are those acquired during the first 2 to 3 years-of-life, in the setting of helplessness and a powerful mediating object. In such a setting, intero- and exteroceptive sensations of distress or comfort activate subcortical survival circuits and primary affects and thus narrowly evoke an unconditioned sense of either a threat to or affirmation of own survival and safety. Primary affects, negative (fear, rage, separation distress) and positive (lust, care, play, seeking) can be conceived as intrinsic brain's value systems that automatically and unconditionally inform animals in real time just how well they are faring in survival, sometimes called “emotional learning”^4^ (Panksepp, 2011). Specifically, positive affects appear to index “comfort zones” that support survival, while negative affects inform about circumstances that may endanger survival; both mediate anticipation of potential safety or danger and, ultimately, increase adaptive fitness of the organism. Emotional learning is a form of implicit memory formed and stored by the brain's emotional memory system (Phelps and LeDoux, 2005). In neonates, the amygdala takes the lead in such emotional learning, but the hippocampus (encoding the semantic-like valence of an event – “aversive” or “rewarding,” not in itself an affect) and mPFC (the thickest region of the PFC at birth, involved in affective processing (reviewed in Hodel, 2018) are also likely involved. The mPFC is activated when mother's speech is directed toward the neonate but stays inactive when the speech is directed toward another adult (Saito et al., 2007) suggestive of early mPFC involvement. In other words, even the earliest experiences may be schematized into implicit schema-affect^5^ complexes. The memories of early object relations are forgotten as specific events but (i) implicitly regulate the relevance and preferential assimilation of new experience as it is being processed by the hippocampus-amygdala dependent learning (Tse et al., 2011), and (ii) represent basic building units, called focal points or attractors, around which functional connectivity networks that underlie the mind self-organize. The trajectories of such interactive self-organization differ significantly as a function of the net-affective valence of experience acquired during phase sensitive formative periods. Predominantly positive experiences facilitate normal brain/mind development (Kolb et al., 2012), while chronic adversity in many instances may re-direct brain/mind trajectories toward personality pathology (Teicher and Samson, 2016). Non-linear self-organizing systems, such as the human brain and mind, tend to follow trajectories toward stable attractors (here positive experiences) as optimal solutions that define the stable “state” of the system. Long before dynamical system theories, Klein (1946) cogently referred to such points of stability as “focal points” for continuous mind integration. Chronic stress during phase sensitive periods affects the organization of prefrontal cortex (PFC) and mPFC (Teicher and Samson, 2016; Czeh et al., 2018), and hippocampus (Luby et al., 2013), likely via epigenetic heightening of the “stress” axis (Dunn et al., 2019). In this context, negative experiences would represent unstable attractors providing only unstable solutions to a complex system thus making it sensitive to minimal internal or external perturbations. Notwithstanding, cognitive framing of activated primary affects may be the initiating force behind mind development in humans (Kernberg, 2014). If so, this process is likely piloted by early object relations.

## The Somatosensory and Limbic Origins of Object Relations

### The Neonate: How Bodily Sensations Initiate Mind Development

The earliest interactive experiences with caregivers evoke mainly bodily sensations, exteroceptive (tactile, olfactory, gustatory), and interoceptive (hunger, pain, thirst, satiety, comfort). Vision and hearing, the senses that inform about the environment the most, have yet to fully develop. Brain systems involved in sensory experiences of one's own body are likely involved in social and emotional processes (Marshall and Meltzoff, 2015). Five-month-old infants manifest implicit body awareness, a fundamental aspect of social perception, as indicated by differing temporal lobe activity during real-time vs. delayed video display of self-initiated face and arm movement; these brain areas are also implicated in body awareness in adults (Filippetti et al., 2015). Cortical sensory-motor body maps might be involved in the basic registration of self–other correspondences which, in turn, facilitates the elementary experience of bidirectionality (Marshall and Meltzoff, 2015). Somatosensory cortices, informed by the insula, brainstem nuclei, and hypothalamus, likely provide direct substrates of mental experiences of body states relevant for survival, e.g., thirst, pain, hunger (Damasio and Carvalho, 2013). In animals, such interoceptive sensations mediate the awareness of some aspects of their own existence (Panksepp, 2011). Evolving cognitive processes, such as selective attention and recognition memory (Reynolds and Romano, 2016), may frame sensory-motor body awareness into the experience of physical boundary with caretakers in cca. 6-month-old infants (aka “self-object boundary”). This nonconscious *physical* “I” represents a mental milestone that delineates the infant's physical entity through the sense of agency^6^ in bidirectional interactions. As Klein (1935, 1946) theorized, object relations are initiated by bodily sensations evoked by earliest interactions with caregivers, via mechanisms of projection and introjection (discussed next).

### Phantasy: The Brain's Innate Code for Primitive Mental Forms

The first attempt to reify projection in neonates is the concept of “phantasy.” Klein (1952) postulated that phantasy is embedded in phylogenetic inheritance and thus predates real experience (Box 1). Phantasy, so the argument goes, is the innate biological code that transforms bodily sensations, biological instincts, and primary affects into *a priori* mental imagery of the inside and outside world (Isaacs, 1948). Early phantasies are bound with bodily sensations and primary affects and these are believed to modulate phantasy into a specific form of imagery (Isaacs, 1948), e.g., pain and fear may create the imagery of paranoia, pleasure the imagery of desire. Phantasy, instincts/affects, and sensations are theorized to mediate projection insofar as they “bring the experiencing mind into contact with external reality” (Isaacs, 1948, p. 92). Visual imagery in dreams reported by congenitally blind individuals (Bértolo et al., 2017) may illustrate phantasy that predates real experience but may not be equated to phantasy as the latter involves *a priori* knowledge beyond visual imagery. Isaacs (1948) posits that such *a priori* knowledge may be inherent in bodily impulses as a vehicle of instinct, in the excitation of an organ, here the mouth (p. 85).

Box 1 **Glossary.**

- **Mind, mental function, psyche, personality**. The terms mind, mental function, and psyche are equivalent as they derive from Latin (*mens)* and Greek *(*ψ*υχ?)* for “mind”. Personality refers to a specific configuration of the mind in any given individual, a unique ontogenetic outcome of the general phylogenetic capacity of human beings. Recent functional neuroimaging of normal subjects (Gratton et al., 2018) shows that functional brain networks have a stable general structure across individuals and also show unique and stable individual differences. Individual variation is most pronounced for top-down control networks, i.e., those that self-organize *via* interactive brain/mind development. Elementary topography of the brain's functional connectivity networks involved in mental faculties (cognition, emotion, and motivation) is present at birth (Bruchhage et al., 2020). The networks are self-organized into spatially segregated but temporally integrated functional units based on person-specific interactive experiences.
- **Episodic and semantic memory. Autonoetic and noetic consciousness**. Episodic memory is contextual, i.e., spatial (“where”), temporal (“when”), and, if applicable, contextual affects evoked by the event. All episodic memories also contain semantic (factual) information (“what happened”). Each type of memory represents different parts of context to form a complete picture. Semantic memory proper refers to information that is independent of the context in which that information was acquired, such as factual knowledge. The dynamic relationship between episodic and semantic memory is discussed throughout this work. Both classes of memory are explicit (conscious). Episodic memories are experienced as autonoetic consciousness, i.e., that the episode happened to the person introspecting his or her memories, only if during the retrieval process a coordinated interplay of psychological capacities (e.g., the sense of self, ownership, subjective temporality, and agency) is integrated into the episode (Klein, 2013). Such involvement of the self makes episodic memories autobiographical (LeDoux and Brown, 2017); otherwise, episodic memory would merely be a complex form of semantic memory (Klein, 2013). Retrieval of semantic memories does not integrate the sense of self in the recollection and such memories are experienced as noetic consciousness of factual knowledge.
- **Taxonomy of affects, emotions/feelings, and sensations**. Affects are complex psycho-physiological states evoked by real-time or recalled exteroceptive activation of subcortical survival and reproductive circuits. Affects consist of subjective conscious experience of emotion, associated ideas and fantasies, and innate physiologic and behavioral response patterns (Auchincloss and Samberg, 2012). Feelings and conscious emotions are interchangeable in our opinion. Interoceptive stimuli, produced by internal organs or by the hypothalamus, are associated with physiologic and behavioral responses which restore homeostasis, consciously experienced as “sensations” of pain, hunger, thirst, etc. The latter may secondarily activate survival- and affect-circuits.
- **Nonconscious vs unconscious**. The term “Nonconscious” is used to refer to the contents of mind not reaching active or passive awareness, while the term “unconscious” is better suited for medical syndromes that include loss of wakefulness, interruption in awareness of oneself and one's surroundings, and unresponsiveness to external stimuli.
- **Phantasy vs. fantasy**. The (controversial) spelling is intended to convey the fundamental difference between the two phenomena, one nonconscious (phantasy) and the other conscious (fantasy), although they may overlap. Klein (Isaacs, 1948) conceived *phantasy* as a phylogenetically inherited, innate biological template to organize survival instincts and unconditioned affects into primitive mental forms. These forms, so the theory goes predate real experience and are sequestered from reality-testing (Isaacs, 1948; Unconscious Phantasy, 2011; Auchincloss and Samberg, 2012). Klein (Unconscious Phantasy, 2011) considered phantasy to operate below the level of *fantasy*, or conscious wishful distortions of reality (colloquially “wishful thinking”) that gratify impulses or solve one's inner problems while reality testing is intact but ignored. As either defensive or gratifying operation, fantasy (wish-fulfillment) may or may not contain elements of deeper, nonconscious phantasies (primitive imaginations of love and aggression with no reality testing). Both phantasy and fantasy have a structuring influence of self- and object-representations (LeDoux and Brown, 2017).
- **Objects in psychoanalysis: real, internal, and external**. In psychoanalysis, the term “object” refers to another person and is contrasted with the self (Auchincloss and Samberg, 2012). Contemporary interpersonal-, relational-, and many object-relation theorists use the term “object” both to refer to another real person and to another person as it is represented in the mind (Auchincloss and Samberg, 2012). In Kleinian psychoanalysis, “external object” refers to a mental representation of another person that is shaped by projection and re-introjection of one's inner cognitive-emotional states, and thus may not accurately represent that person (Auchincloss and Samberg, 2012). “Internal object” refers to a mental representation of another person that one invests with drives and affects, aka “significant objects,” whose interactive impact has been internalized and continues its influence on the mind from within. A complex interplay continues throughout life between internal objects and those in the real world as they are represented in one's mind (Internal Objects, 2011), a somewhat solipsistic but a plausible approach to the reality of external objects in the mind of the beholder.

#### Science of Phantasy: The Plausibility Argument Only

The concept of phantasy awaits scientific validation. It is however plausible that the origination of human mind has a head-start via some innate biological code that *predictably* transforms physical sensations, biological instincts, and unconditioned affects into rudimentary mental forms. Randomness at early stages of mind development seems implausible from an evolutionary perspective. In the absence of direct evidence, Ogden (1984) reviews examples of psychological “deep structures” acting as innate biological codes that organize perception and early learning and might support the concept of phantasy by analogy. One such code is Piaget's pre-existing “innate systems” in which children organize thinking into structured clusters of concepts. However, this and other examples from linguistics and anthropology (Ogden, 1984, p. 502–503) concern an advanced brain organization relative to neonates, and might reflect the intrinsic concept-making propensity of the brain's convergence regions^7^ (LeDoux, 2002; Wang et al., 2012) which are immature but fast developing since the latter half of infancy and toddlerhood (reviewed in Merve Çikili, 2018). In contrast, phantasy in neonates might arise from information processing in the limbic system (instincts, affects) and sensory-motor association cortices (intero- and exteroceptive sensations), both operational at birth. If so, such processing might generate a type of *a priori* imagery of the outside world, perhaps reflecting archaic mental forms associated with activated biological instincts and unconditioned affects.

### Introjection: Taking the Outside World Into the Mind

The psychoanalytic term “introjection”^8^ metaphorizes the process whereby the mind is modified by real or phantasized aspects of the outside world that are “taken into the mind” as mental representations of that world (Auchincloss and Samberg, 2012, Box 1). In neonates, so the theory goes, upon activation by instincts and affects, projected imagery of phantasy is tested against actual experience evoked by the interaction (Isaacs, 1948). The resulting blend is introjected as an object-representation, at first dichotomized by evoked affects into “horrifying” (fear for survival in the context of hunger, pain, etc.) or “idealized” (affirmation of survival in the context of comfort, safety, etc.). Such object-representation becomes an “internal object” that continues its influence on the mind from within (Box 1). Embedded in object-representations are self-representations that are believed to arise from the neonate's non-conscious detection of own activated instincts, sensations, and affects being linked with an outside presence^9^. A self-representation thus shares affective valence and develops in tandem with object-representation of which it is initially a part (Jacobson, 1964).

#### Neuroscience of Introjection

Object relation theorists pioneered the idea that introjection, a mental portrait of the outside world, must have a neural substrate in perception and memory (Riviere, 1952), both processes of “primary autonomy” independent from instinctual drives (Hartmann et al., 1964). Introjection allows the brain/mind to record the history of real or perceived interactions between the individual and the world of objects, and as such plays a major role in mental life throughout the life-cycle, from early self- and object-representations to one's acquisition of culture (Auchincloss and Samberg, 2012). As noted, object relations arise from early survival- and safety-level experiences with caretakers and thus evoke peak unconditioned affects (fear, rage, pleasure, safety). The biological basis of such learning likely involves memory, here affect-enhanced episodic memory, the brain faculty by which self-related events are encoded as memory traces or engrams^10^ and operate inside the mind as a sustained presence. The incidence of experiences narrowly related to survival and safety decreases with increasing cognitive and physical autonomy. Emotion-enhanced episodic memories continue after childhood, but become mostly concerned with experiences that either promote or frustrate one's current goals (Talmi et al., 2019).

## Partial Object Relations

### The Theory

From birth to about 6 months of life, neonates and infants suffer a great deal of anxiety about own survival and safety, first caused by the trauma experienced during delivery, and postnatally by occasional but inevitable hunger, pain, frustration of instinctual needs, etc. Per Klein (1935, 1946) such distressful experiences are phantasized to be threats of annihilation by some outside invasive persecutor – conceptualized as phantasy of “Not I” in Kleinian psychoanalysis. Phantasy is molded by activated unconditioned affects and survival-instincts into paranoid fears for own safety with defensive impulses to destroy the threat (“paranoid-schizoid position”) (Klein, 1946). The projected destructive impulses, at first directed at the breast and later at the mother, are partly re-introjected and become the internal persecuting “bad object” (Klein, 1946). Positive experiences (safety, pleasure, comfort – or phantasy of “I” in Kleinian psychoanalysis) are split-off from their negative counterparts and thereby protected from destruction from within (“partial object relations”). Klein (1946, p. 99) uses the phrase “division of love and hate” to describe the unrealistic but purposeful split between contradictory experiences toward the same object. Self-representations derived from the interactions are also dichotomized, gratified *or* frustrated, for they share the affective valence with gratifying *or* frustrating object-representations (Jacobson, 1964). Klein (1935, 1946) thought of early splitting as a normal and purely mental mechanism that is essential for continuous mind organization around the focal point of positive experiences. During normal development, splitting is “binary,” or consisting of two contrasting wholes, in which case the predominant “good” self- and object-representations serve as the foundation for less frequent “bad” episodic experiences to be added and neutralized as exceptions. Prolonged uncontrollable stress may perpetuate pathological persistence of dichotomized self- and object-representations, which, by means of multiple splitting, remain unsystematized and co-exist as split off fragments, each a unipolar unit of experience (“bad” or “good”) about one's own self and others. Specifically, chronic severe stress in neonates and young infants may disrupt the intrinsic concept-making ability of mPFC, an effect reported in rodents (Czeh et al., 2018) and may account for multiple splitting of partial object relations in severe cases of personality disorder (Kernberg, 1975). In less severe cases, splitting may reach binarity (“good” vs. “bad” as contrasting wholes) but experience remains polarized due to stress-induced disruption of prefrontal cognitive integrations (reviewed in Teicher and Samson, 2016).

### Neuroscience of Partial Object Relations: Affective Dichotomization of Sensory Stimuli as the First Organizer of the Mind

Studies of contextual fear conditioning in rodents use recombinant DNA (Gossen and Bujard, 1992) that enables activity-dependent gene expression in neurons (Reijmers et al., 2007)), *in vivo* imaging of calcium-dependent neuronal activity, retrograde pathway tracing, gain of function manipulation, and optogenetics (Redondo et al., 2014; Kim et al., 2016; Kitamura et al., 2017). This methodology has demonstrated how affect-enhanced episodic memories form. The semantic-like content (i.e., “what” - an aversive or rewarding event, not in itself an affect)^11^, spatial context (“where”) are processed by distributed neocortical regions and the hippocampus (Howard, 2017; Tonegawa et al., 2018). However, a full temporal context (“when”) is not available in infancy. Subjective awareness of time is a network phenomenon that appears to involve left lateral parietal and left frontal cortices, cerebellum and thalamus (Nyberg et al., 2010), although the hippocampus might some ability to form temporal order (Eichenbaum, 2014). Subjective time is mentally constructed by adding temporal dimension to episodic memories (past and future thinking) (Nyberg et al., 2010) and is aided by the perceptual vividity and the ease of extracting information from the stimulus (Matthews and Meck, 2016). Temporal order of events appears to be assembled in non-conscious working memory (Trübutschek et al., 2019), before being re-represented in conscious working memory, and thus can be attained in mid-to-late toddlerhood when such cognitive higher-order processing is sufficiently developed.

The hippocampus forms an index of neural activities that were present during the actual experience (Teyler and DiScenna, 1986). The contextual affect is engramized in the amygdala (Kitamura et al., 2017) by neurons genetically programmed to encode only rewarding *or* frustrating affects (Kim et al., 2016). As they are being encoded via synaptic plasticity, the hippocampal engrams engage mPFC to form its corresponding indexing engram, at first silent but within several weeks of continuing hippocampal input^12^, mature to become the active memory of the episode (Kitamura et al., 2017). The mPFC engram cells are believed to enable amalgamation of distributed component engrams of multimodal information into a coherent episode (Tonegawa et al., 2018), now “systems-consolidated.” The hippocampal originals then become silent, in the sense that they show a decreased density of dendritic spines and can be reactivated only by optogeneitc stimulation, but not by natural clues; whether the hippocampus eventually loses the memory information is unknown (Tonegawa et al., 2018). The amygdala also provides input during the formation of the mPFC engrams but unlike the hippocampus, it remains active in providing the affect-memory of the experience (Kitamura et al., 2017). The process of contextual fear memory is described here but a similar process is likely for reward-enhanced memories both types qualifying as episodic-like memories (Phelps and LeDoux, 2005; Kitamura et al., 2017). Extrapolated to human infants, contradictory affects evoked by frustrating vs. rewarding interactions with caretakers are likely encoded by separate groups of amygdala neurons dedicated to only one of the valences. As each group provides affect-information about its respective experience, self- and object-representations derived from these episodes are dichotomized by affects into either rewarding or frustrating^13^. The psychoanalytic concept of splitting between affectively dichotomized self- and object-representations may therefore be hardwired in the amygdala. Psychoanalysis and neuroscience appear to describe the same phenomenon from different angles, functional and neuroanatomical, respectively.

## Integrated Object Relations: Cognitive Reconciliation of Affective Dichotomies …and Beyond

Two parallel developments mark the transition from partial to integrated object relations, a gradual cognitive process evolving from roughly 6 months to 2–3-years-of-life^14^, when self- and object-constancy are established. The infant/child becomes increasingly adept at:

- integrating dichotomized experience into a realistic cognitive-affective frame whereby significant objects (and the related sense of self) can be experienced as *both* gratifying *and* frustrating) and
- mentally differentiating self- from object-representations; such *mental* boundary toward others allows the sense of self to be internally maintained, and increasingly independent from caretaker's affects and actions.

The phenomenology and mechanisms of whole object relations are discussed in more detail below.

### Neuroscience of Integration of Dichotomized Experience: Schematization of Episodic Memories

#### Episodic-to-Semantic Memory Conversion: Generalization of Knowledge From Specific Experiences

Mental functioning would be inefficient, if not impossible, if episodic memories retained the level of external contextual detail present at encoding. In “Funes the Memorious” by Borges (1998), a fictional character Ireneo Funes remembers absolutely every single detail of all episodic experiences. Thus, Funes has no need for generalizations, the essence of thinking, and his remarkable capability becomes a cognitive limitation.

All episodic memory is first encoded by synaptic plasticity in the hippocampus-amygdala memory system. As noted, the hippocampus forms an index of neocortical activities that encode individual components of multimodal sensory experience (Teyler and DiScenna, 1986). The amygdala encodes the affect-memory of the experience (Kitamura et al., 2017). The mechanism of systems-consolidation of episodic memory is contentious. Multiple trace theory posits that the hippocampus records multiple traces of related episodes, and as the number of traces increases so too does the likelihood that the episode is accessible to remote recall (reviewed in Kandel et al., 2014). Alternatively, recent findings show how the mPFC, engaged by inputs from the hippocampus and amygdala, consolidates long-term episodic memories while the hippocampal engrams become silent (reviewed in Tonegawa et al., 2018). Whether remote episodic memories continue to depend on hippocampus for context retrieval is also a point of contention. These questions, important for memory research, are not central to this discussion. What seems to be certain is that multiple related episodes are extracted into a gist-like neocortical schema based on overlaps, statistical regularities, and abstractions, aka “schema-learning” or “episodic-to-semantic conversion” (Kandel et al., 2014; Tonegawa et al., 2018). Gist-like neocortical schemas are independent of specific learning episodes, as their main function is to extract abstract knowledge from specific past experiences (Wiltgen and Silva, 2007) that can be generalized across similar-but-novel situations that may arise in the future (details in Box 3).

#### Intrinsic Propensity for Schema Learning and Rudimentary Working Memory Are Plausible Mechanisms of Integrated Object Relations

In the context of good-enough parenting^15^ and/or heritably tempered affect reactivity, integration of affectively dichotomized self- and object- representations results in each having a predominantly positive valence, which tends to remain stable despite occasional frustrations. Such “self-constancy” (Mahler et al., 1975) and “object-constancy” (Hartmann, 1952) evolve in tandem from about 6 months to 2-3 years-of-age through co-determined processes (Auchincloss and Samberg, 2012). Attainment of self- and object-constancy is a developmental milestone essential for further progression of mind development and social relations.

Anatomically segregated groups of amygdala neurons are genetically programmed to encode only one affective valence of episodic memories (Kim et al., 2016). Thus, the dichotomies inherent to partial object relations cannot be integrated *in situ*, only by higher-order cognitive synthesis. Intrinsic processing within mPFC is known to be involved in rule and categorization memories, including the formation of schematic frameworks for episodic memories (Wang et al., 2012). Klein (1935, 1946) observed the emergence of integrated object relations around 6-months-of-life. If so, this introductory integration of polarized experience could be piloted by the mentioned intrinsic concept-making propensity of the mPFC. The process is likely facilitated by emerging recognition memory and selective attention (Reynolds and Romano, 2016). The outcome of such processing may be an implicit, gist-like schematization of related interactive episodes, i.e., that primary affects recognized as own^16^ may alternate between positive and negative across contradictory experiences with the same object. Individual components of early care tend to be positively correlated. A good-enough caretaker generally delivers adequate but not perfectly good care and *vice versa*. As the hippocampus provides the contextual, semantic-like valence of enhanced episodic memories (Eichenbaum, 2017; Tonegawa et al., 2018), schematized related events likely coalesce into a net-valence based on regularities in care (*usual* distress vs. *usual* comfort), ultimately a net-experience of threat to or assurance of survival. Implicit integration into *good-and-bad* self- and object-representations therefore means a predominance of one valence. The net-rewarding valence of early integrated object relations may implicitly dampen negative affects aroused by frustrating interactions with the same object.

Unconditioned survival-level experiences of neonates and infants evolve to bilateral participation in danger/safety learning in toddlers. The willful role of the caretaker as the source of reward and frustration can be recognized by toddlers, likely reflecting the ongoing development of selective attention and working memory. These are two functionally inter-dependent cognitive processes (Marchetti, 2014) are closely tied to the biological maturation of the PFC, both are network phenomena, and both are rudimentary but fast developing after roughly 6–8 months of age (Reynolds and Romano, 2016).

Whenever a self- and object-representation of negative valence is activated, it can now be balanced in working memory by its positive opposite during the “reparation” process with a good-enough parent (to be discussed). The mPFC is essential to providing such emotional information to working memory (Smith et al., 2018). Occasional negative experiences with the same significant object can still be assimilated but neutralized as exceptions. An interesting possibility is that such cognitive-affective balancing may occur within non-conscious working memory where neural engrams are “higher-order represented” by cognitive processing, but are still non-conscious (LeDoux and Brown, 2017). Non-conscious working memory can be conceived as a hidden layer of the mind because its neural input is amenable to modifications by covert yet teleological cognitive influences (Bergstrom and Eriksson, 2018) before its output is re-represented in conscious working memory (LeDoux and Brown, 2017). Specifically, cognitive integration of dichotomies may involve attention-mediated modifications of synaptic-weights and/or oscillatory phase-locking that modify functional connectiviy. Both may influence the neural input to working memory representing affectively opposite valences across interactive episodes. The process of integration is likely non-conscious, as is the case with most other cognitive processing (Kihlstrom, 1987). However, the outcome (output), i.e., the experience of self- and object-constancy, may reach phenomenal consciousness in toddlers, as first-person experience “for there to be something that it is like for one” (LeDoux and Brown, 2017), here the predominantly positive relationship with the caretaker.

Neither the survival-level experiences of infancy nor the safety-danger experiences of toddlerhood remain as memories of specific events. The brain structures and large-scale networks involved in long term memory, e.g., the hippocampus and PFC, among others (Wheeler et al., 2013) are not sufficiently mature in infants and toddlers. Moreover, the subsystem of self, which fortifies episodic memory by self-referenced cognition, has yet to fully self-organize in toddlers. Self-reference provides a form of associative glue for perception, memory, and decision-making and, through this, acts as a “central mechanism in information processing” (Sui and Humphreys, 2015, p. 719).

### Mental Separation of Self- From Object-Representations

In parallel with the integration of contradictory self- and object-representations, the experience of self, initially embedded in object-representations, achieves phenomenological autonomy to emerge as independent experience. Such *mental* differentiation of self-representations from internalized object-representations is a milestone first recognized around 18–24 months-of-age, an approximation based on Mahler et al. (1975). The PFC develops most rapidly during the first 24 months of life (review in Hodel, 2018) thereby providing neuro-cognitive mechanisms for such differentiation to emerge. The recently formulated HOTEC (LeDoux and Brown, 2017; Brown et al., 2019) has been challenged (Naccache, 2018). The HOTEC, however, provides a cogent account of the development of the sense of self by cognitive processing of semanticized episodic events, at the core of which is the implicit self-schema extracted from early object relations.

#### The Sense of Self: From Non-conscious Neural Engrams to Self-Awareness

As LeDoux and Brown (2017) summarize, adult self-schema includes (i) autobiographical episodic memories, (ii) semantic memory of one's identity (“who am I”), (iii) semanticized episodic memories across related interactive episodes converted into semantic knowledge of one's own personality traits (Klein, 2013), i.e., how one *usually* feels, acts, and thinks in particular situations (LeDoux and Brown, 2017), and (iv) one's body-schema, i.e., static and kinetic state of the body as well as its functional integrity (Mendoza, 2011; Morasso et al., 2015). These components of a self-schema exist as non-conscious engrams and body-sensing neural systems and are thus non-mental, first-order *neural* states, until represented by maturing cognitive networks in working memory, when neural encodings become higher-order *mental* states (LeDoux and Brown, 2017). Such higher order cognitive representation in working memory unifies the subjective background information about the physical self with mental experience of the active working self into an “embodied self” (Kernberg, 2014). The debate whether neural memory engrams and subcortical affect circuits per se can generate subjective conscious experiences that does not involve cognitive representation is ongoing (“first-order” vs “higher-order theory of emotional consciousness”). We have adopted the HOTEC (as seen in Box 2) but acknowledge alternative views.

Box 2 **Self and conscious emotions: from neural engrams to enhanced episodic memories of which one is a self-aware feeling part.**

As LeDoux and Brown (2017) summarize, adult self-schema includes (i) memories of self-related episodic events, (ii) semantic memory of one's identity^2^, (iii) semantic memory about one's own personality traits, i.e., how one usually feels, acts, and thinks in particular situations (Klein, 2013), and (iv) one's body-schema, i.e., static and kinetic state of the body as a whole and its individual parts, such as location and orientation of various parts and relative motion in space and time, as well as functional integrity (Mendoza, 2011). Complex inter-connectedness between self and autobiographical knowledge is the self-memory system (Conway, 2005): different types of memory provide data that constrain the working self, defined as a set of active personal goals and self-images in particular social-communicative interactions (Guerini et al., 2019), but in turn memory is also reciprocally controlled for accessibility by the working self (Conway, 2005). Again borrowing from LeDoux and Brown (2017), conscious emotions of which one is a self-aware part (i.e., autonoetic consciousness of self and emotions) are tied to representation of the self as part of a higher-order state that constitutes the felt experience [“no self-no emotion” (LeDoux and Brown, 2017, p. E2023)]. Conscious emotions are born by the same sequence of cognitive HOR, HOROR, and HOR of HOROR although with a different neural input from: (i) engramized emotion schemas (memorized facts about a particular affect-provoking situation, but not itself an emotion), (ii) activated subcortical survival circuits and affects (evoked affects are cognitively represented as conscious emotion in working memory), (iii) the relevant self-schema (i.e., how one usually feels, thinks, and acts in that particular situation), and iv) autobiographical memories of similar events (LeDoux and Brown, 2017).

Borrowing descriptors from LeDoux and Brown (2017), the engramized self-schema becomes a conscious sense of self through a series of higher order representations (HOR) and re-representations (HOR of HOR, or HOROR) by the general network of cognition in working memory (Box 4). Each new representation is a higher-order cognitive reflection of the lower-order representation, figuratively a “recognizer of recognizers” (Edelman and Mountcastle, 1982). The first HOR of the semantic self-schema (self-HOR) within non-conscious working memory remains non-conscious while still allowing for an autonoetic conscious state of the self to result from the re-representation, self-HOROR, in conscious working memory (LeDoux and Brown, 2017, p. E2019–2020). The re-representation gives rise to a phenomenally conscious experience of self with minimal inner awareness. The self is only passively noticed as the first *person* subjective experience of “knowing what is it like” for me…(the taste of an apple)… and *phenomenal quality* of experience (e.g., the taste of apples vs. the smell of a rose) (LeDoux and Brown, 2017). The background presence of first person phenomenal experience establishes a *mental* boundary with objects as they are represented in the mind, figuratively a *mental* “I” coexisting with others. Reflecting this milestone, 3–4 year old children-peers are able to “keep the secret” for each other (which involves understanding boundaries between information that one possesses and information that parents possess (Gordon et al., 2014) or tell a lie (reflecting their emerging understanding of the subjective nature of beliefs) (Evans and Lee, 2013). Both phenomena are suggestive of the child's awareness of the impenetrability of his or her mind. First mental clashes with the world also emerge around this age, aka “the terrible twos,” coinciding with the emergence of “autonomy and will” in Erikson's (1959) terms. An actively introspective, self-aware self emerges through yet another re-representation of the phenomenally experienced self, a HOR of self-HOROR (LeDoux and Brown, 2017). Capacity for introspection has been demonstrated in 3–4 year-old-children and is thought to be a key prerequisite for the theory of mind of others (Gonzales et al., 2018). The mechanism of higher order (re)representations in working memory is unknown. Some possibilities are reviewed in Box 4.

Box 3 **Schema-learning: extraction of general concepts from naturally and/or affectively related specific experiences.**

Schematized experience (schemas), defined as associated knowledge structures that overarch related episodic memories, may involve *de novo processing* by the mPFC which is suggestive of a new schema-engram, aka “episodic-to-semantic-conversion” (Tonegawa et al., 2018). Of note, neither mPFC engram cells nor circuitry for a semantic memory encoding a schema for related episodic memories have been found (Tonegawa et al., 2018). Alternatively, schema-learning may arise from the *natural tendency* of episodic memories to semanticize over time, i.e., to lose spatio-temporal context-specificity (“when” and “where”) while semantic information (“what happened”) and contextual affects (“how it felt”) remain. Here, “episodic-to-semantic-conversion” naturally takes place within mPFC (Tonegawa et al., 2018), perhaps reflecting the process of systems-consolidation of memory (Wiltgen and Silva, 2007; Dudai, 2012). Neurophysiological and epigenetic processes involving mPFC and hippocampus in schema-learning and self-reinforcing expansion have been described in animals (Tse et al., 2011; Wang et al., 2012). A similar pattern in humans, involving crosstalk^1^ between the hippocampus and ventromedial PFC (vmPFC) has been established for schema-forming, self-reinforcing schema-expansion, and schema-alteration (van Kesteren et al., 2010, 2012, 2016; van Kesteren and Meeter, 2020). The hippocampus forms an index of attending neocortical activities involved in encoding individual components of contextually reach memory and connects with amygdala-encoded contextual affects evoked by the episode (Tonegawa et al., 2018). A corresponding index is generated by the mPFC during system-consolidation of episodic memories, a process guided by hippocampus (Tonegawa et al., 2018), while the amygdala continues to provide input about affects associated with the-memories (Kitamura et al., 2017). Reinstatement (reactivation) of a previously learned episodic memory during new hippocampal-amygdala encoding may facilitate across-event generalizations and construction of schemas (van Kesteren et al., 2016). Semantic (natural) or affective congruence with an existing schema, perhaps determined by mPFC-amygdala crosstalk, my trigger such reactivations which are thought to be executed by the hippocampus (Preston and Eichenbaum, 2013). The mPFC may detect an activated schema and update it (van Kesteren et al., 2012; Preston and Eichenbaum, 2013) by integrating new or overlapping information into the schema. These updates are achieved by re-consolidation during sleep and rest [reviewed in van Kesteren et al. (2010) and van Kesteren et al. (2012)] and may result in re-construction (expansion or alteration) of the existing schema. Once developed, a schema might not be expanded only within mPFC, but the hippocampus might provide “semantic-like” generalization rather than “episodic-like” learning to add to the existing cortical semantic schema (Tse et al., 2011).

Box 4 **Large scale functional brain networks dynamically interact to deliver various facets of cognition.**

The general network of cognition (LeDoux and Brown, 2017) encompasses a number of partially overlapping large-scale functional connectivity networks (and their composite subnetworks) that spread across parietal and medial and lateral frontal regions and dynamically interact to produce cognition, e.g., central executive network engaged in higher-order cognitive and attentional control, salience network that is thought to mediate attention to monitor the salience of external inputs and internal brain events, default mode network that is thought to comprise an integrated system for autobiographical, self-monitoring, and social cognitive function, among other networks (Bressler and Menon, 2010). The mechanism of higher order (re)representations in working memory is unknown but could involve rapid transitions in activity silent neural states, such as short-term synaptic weight changes induced by periodic spiking-bursts within cognitive networks perhaps involving calcium dynamics. (Miller et al., 2018). As a complementary mechanism, frequency-specific oscillatory coherence could mediate temporary shifts in functional connectivity by phase aligning periods of excitability to maximize the opportunity for information transfer (and phase misaligning to suppress communication) (Stokes, 2015). Such hidden state transitions appear to flexibly configure memory networks for memory-guided behavior and dissolve them equally fast to allow forgetting (Wolff et al., 2017).

### Beyond the Integration of Dichotomies: Working Through the Anxieties of Depressive Position

Cognitive integration of dichotomized object-representations has been termed “depressive position” by Klein (1935, 1946), because the dyadic object comes to be experienced by the infant as whole object, who can be frustrating but is generally rewarding and is thus idealized; this, in turn, generates depressive fears that the object could be lost. Achievement of depressive position creates a number of new experiences that need to be “worked through” (psychoanalytic phrase for “resolved”), a gradual process from about 6-months to about 3-years-of-life. Paranoid fears about one's own safety are now accompanied by anxieties about the safety of the object, whose absence, for example, is now experienced as loss rather than a persecutory attack, as in partial object relations (Etherington, 2020). Fear of losing the object and guilt about one's aggressive impulses that damage internal self- and object-representations are the most important of these new experiences. In times of frustration, fear and guilt activate the drive for reparation in the child (Klein, 1933). Compassionate reassurance by a good-enough mother restores and strengthens the child's positive inner representations of both. During the working-through period, mediated by good-enough parenting, guilt-driven anxieties about the safety of the object mature into a genuine concern for the welfare of the object, joy in giving and mutual exchanges, empathy and reciprocity, while the destructive envy of the object's powers evolves into gratitude for care and support (Klein, 1957). At this point, around 3 years of age, a stable good internal object, corresponding to self-regulating object-constancy, a successful outcome of the separation-individuation process (Mahler et al., 1975), and an autonomous self, mainly internally defined and maintained (Hartmann, 1950; Kernberg, 1975), are established. Both attainments set the stage for stabilization of affects across contradictory interactions (affect-constancy). Successfully working through sadness, guilt, envy, jealousy, Oedipal issues, and anxieties of depressive position, facilitated by support from significant objects, is a prerequisite for the capacity for mature love (guilt and sadness are an inherent part of love and deepen it profoundly) (Klein and Riviere, 1937) and healthy interpersonal and social mental life. Notwithstanding, working through the anxieties of depressive position continues over lifetime during personal crises, stress, or challenges in significant relationships (Klein, 1935, 1946). Unresolved childhood anxieties of depressive position may increase one's vulnerability to maladaptive core beliefs, depression and anxiety in particular, despite integrated dichotomies of partial object relations (Box 6). The concept of depressive position is considered by many to be the finest of Klein's contributions to understanding the human mind.

#### Neuroscience of Successful “Working Through”

Interactive self-organization of the PFC, a key hub in the brain's general network of cognition are a plausible neural substrate for the successful “working through” the anxieties of depressive position, culminating in an internally defined and maintained sense of self and cognitive-emotional constancy of significant objects. The general network of cognition (LeDoux and Brown, 2017) encompasses a number of partially overlapping functional connectivity networks (and their composite subnetworks) that spread across medial and lateral parietal and frontal regions, and dynamically interact to produce various facets of cognition (Bressler and Menon, 2010). These include, among others, central executive network engaged in working memory and top-down attentional control (Miller et al., 2018), salience network that is thought to mediate attention to monitor the salience of external inputs and internal brain events as well as to recruit relevant functional networks (Goulden et al., 2014), default mode network that is thought to comprise an integrated system for autobiographical, self-monitoring, and social cognitive function (Bressler and Menon, 2010). The maturing general network of cognition has been implicated in the emerging capacity for working memory and executive top-down control of subcortical affect reactivity (Miller et al., 2018), internally motivated selective attention (Corbetta et al., 2008), the overlapping capacities for the theory of mind (Corbetta et al., 2008), cognitive empathy, helping behaviors, moral reasoning (Preston and de Waal, 2002), mentalizing (Luyten and Fonagy, 2015), self-referenced cognition (Sui and Humphreys, 2015), and conscious emotion (LeDoux and Brown, 2017), among others. These advanced mental capacities co-emerge during the integration of dichotomized object relations and mediate working through guilt and other anxieties of depressive position created by this integration.

### The Self: A Composite of Coexisting More-and-Less Integrated Object Relations

Around 3 years-of-age, social life begins to expand to include other significant objects, the father and selective family, later on selective friends and peers, romantic partners, work, among others. These significant relationships are first processed for safety and acceptance through the master prism of early object relations, but also tend to add specific cognitive-affective generalizations to the sense of self and the world of objects. Under normal circumstances, integrated object relations expand to include other significant relationships in the context of stable self-esteem and healthy trust. In personality disorder, partial object relations expand to include experiences of rejection in the context of one's own fragility, endangerment, and general distrust of others. Reflecting the influence of multiple objects of variable significance in different personal domains and at different levels of mental integration of both parties, practically everyone's mind harbors a mosaic of more and less integrated mental representations of themselves and others (Auchincloss and Samberg, 2012). As Fairbairn (1954) observed in practice, multiple coexisting units of the unified experience of self arise from an element of self-engaged in affect-energized relationship with an element of a significant object. In other words, even within the same significant relationship, there can be more or less mature self- and object representations. It is the pervasiveness of what is polarized or balanced that define normalcy vs. pathology of object relations in any given individual (Svrakic and Divac-Jovanovic, 2019). If there was a single network that would represent the neural substrate of the sense of self, the DMN and the dorsal mPFC as one its key hubs are the prime candidates (Box 5).

Box 5 **The default mode network (DMN) could be the neural substrate of the self.**

The DMN is a functional connectivity network comprised of temporally integrated activity in distributed brain regions (nodes) involved in numerous cognitive functions, such as mPFC (social cognitive processes related to self and others, sense of agency Renes et al., 2014), posterior cingulate cortex / precuneus (autobiographical memory, self-referential processes) (Fransson and Marrelec, 2008), medial temporal lobe (episodic memory), angular gyrus (semantic memory), among others (Bressler and Menon, 2010). The DMN exhibits strong low-frequency oscillations coherent during resting state and is deactivated, although not completely, during attention-demanding goal directed tasks (Sormaz et al., 2018). The brain defaults to DMN during wakeful rest, disengaged from the external world but engaged in social working memory (e.g., theory of mind of others, empathy, moral reasoning, and social evaluations), reflecting upon one's own autobiographical history, traits, and emotions, and projecting oneself into the future (“internal mentation”) …a form of “travel by mental simulation through our past, future, and the minds of others”… (Andrews-Hanna, 2012, p. 13). If there was a single network that would represent the neural substrate of the sense of self, the DMN in general, the dorsal mPFC as one its key hubs in particular, are the prime candidates (Gusnard et al., 2001). The incomplete deactivation of the DMN during attention-demanding goal-directed tasks (Sormaz et al., 2018) might be the mechanism of self-referential cognition and emotion mediating the continuous representation of self in ongoing mental activities.

## Early Behavioral Manifestations of Object Relations

The master schema of early object relations underlies implicit predictions of outcomes and automatized action programs in significant relationships, as discussed. The earliest example of this implicit guidance may be observed as attachment patterns (Benoit, 2004). Attachment refers to preconceptual internal models of behaviors related to safety and protection within a specific and circumscribed aspect of the relationship between a child and caregiver (Benoit, 2004). Object relations and attachment patterns are coincidental (both are observed in 6 to 24-month old infants/toddlers) (Klein, 1935, 1946; Benoit, 2004), both correlate with the quality of parenting and/or heritable affect reactivity (Kernberg, 1975; Benoit, 2004), and both are implicit and preconceptual; the above is suggestive of linear relatedness between early object relations and attachment patterns. The master schema of *whole* object relations determines implicit anticipation of a reassuring caregiver's response to the child's negative emotions (secure attachment). The master schema of *partial* object relations determines implicit anticipation of either outright rejection, unpredictable/varied, or atypical/distorted caregiver responses to the child's distress (van Ijzendoorn et al., 1999; Benoit, 2004). The insecurely attached infant/child builds implicit action programs to deal with negative emotions in the presence of bad-enough caretakers, observable as pathology of attachment. The latter is classified as insecure-avoidance toward rejecting parents, insecure-resistance with display of amplified distress toward unpredictable parents, and disorganized, bizarre or contradictory behaviors in the context of distorted or atypical parenting (van Ijzendoorn et al., 1999; Benoit, 2004). Attachment pathology is predictive of poor social and emotional outcomes (Benoit, 2004), including personality disorder (Levy et al., 2005), the latter especially severe with antecedent disorganized attachment (Beeney et al., 2017).

### Adaptive Value of Whole Object Relations: Balancing the Mind and Behavior

Affective dichotomization of sensory stimuli is hardwired in the normal anatomy and function of the amygdala (Kim et al., 2016). Such hardwired polarization is evolutionarily important as it enables rapid either-or responses when subcortical affects and survival instincts guide behavior (e.g., freeze-fight-or-flight). The development of PFC, one of the key hubs in attention-reorienting (Corbetta et al., 2008) and working memory networks (Wang et al., 2006), has enabled humans to carry out internally chosen goal-directed behavior and avoid affective distractions by maintaining attention, planning, and volition, aka “executive function.” The latter is one of the two main functions of working memory, the other is to maintain information “online” for comparisons, recognitions, and contrasts (Miller et al., 2018). In the vast majority of human personal and interpersonal emotional situations, balanced and reflective rather than extremized and reflexive mind and behavior are adaptively advantageous, while amygdala-driven reactions remain important for emergencies.

## Neuroscience of Key Psychoanalytic Postulates About Object Relations

### Object Relations Are the Basic Building Units of the Mind

Psychoanalysis postulates that “all psychological experience, from the most fleeting fantasy to the most stable structure, is organized by object relations… the basic units of all experience” (Auchincloss and Samberg, 2012, p.175)^17^. Cognitive-, cellular-, and systems-neuroscience of schematized episodic experience in humans (van Kesteren et al., 2016; van Kesteren and Meeter, 2020) and rodents (Tse et al., 2011; Wang et al., 2012) provide a potential mechanism for this theorizing. Schematic frameworks for survival- and safety-level experiences of infancy and toddlerhood appear to be formed and stored in the mPFC while the amygdala continues to provide input about contextual affect-memories (Kitamura et al., 2017), as discussed. Together these implicit cortical-subcortical encodings constitute the primordial schema-affect template of the mind. Self-reinforcing patterns of schema expansion and updates are similar for rodents and humans (Box 3). The implicit schema-affect template *itself* may not be accessible to representation by cognitive networks of which it is the integral part and may remain something of an epistemic quandary (Solms, 2015). Nonetheless, it profoundly influences the subsequent self-organization of functional connectivity networks that dynamically interact to deliver mental faculties of cognition, motivation, and emotion (Zorumski and Rubin, 2011). The mental faculties can be thought of as the “tools of the mind” that, each from a different angle but acting in concert, enable the mind to communicate with itself, the inner and the outer world, and to change and/or adapt to both. The second avenue of mind development involves a sequential differentiation of its subsystems, e.g., temperament traits, the experience of self, identity, character traits, and moral values (Svrakic and Divac-Jovanovic, 2019). The subsystems can be thought of as semi-stable functional coalitions of relevant cognition, motivation, and emotion that emerge in a predictable order, temperament fist – character and moral values last, to meet increasingly complex adaptive demands; each subsystem contributes a unique adaptive function to the overall adaptive fitness of the mind (Svrakic and Divac-Jovanovic, 2019). The inaugural template of early object relations harbors the rudiments of mental faculties, i.e., the semantic-like content – rewarding or aversive event, the associated affect of fear or pleasure, and the motivation to self-protect or engage, all implicit, i.e., non-conscious, and non-declarative^18^. As one of the main convergence zones in the brain, the mPFC is a key hub in multiple functional connectivity networks that underlie mental faculties. The schema-affect template of early object relations is therefore likely to influence interactive self-organization of these networks from within, acting as a non-conscious operating system that determines the relevance of future experience upon which these networks are built. Such personalized self-organization of the brain/mind adds a person-specific component to the relatively stable general structure of functional brain networks (Gratton et al., 2018), ultimately responsible for the mental uniqueness of the individual or “personality.” Each mental faculty and each subsystem of the mind are thus more or less distantly related to early object relations. For example, rudiments of empathy, fairness, and reciprocity as forerunners of character traits and moral values are detected in securely attached infants and toddlers (Wynn and Bloom, 2013) presumed to have achieved self- and object-constancy. These forerunners are later amended by explicit socio-ethical norms to facilitate social adaptation.

### Object Relations Are Dynamic and Reversible

Klein (1935, 1946) describes paranoid-schizoid and depressive positions to differentiate developmental *stages* that the child progresses through, from *positions*, or “ways of being,” that humans oscillate between throughout development and into adult life (Etherington, 2020). Klein (1935, 1946) observed reversible shifts from paranoid-schizoid position to depressive position and *vice versa* occurring over lifetime in response to stress, loss, or personal crises and the resolutions of anxiety and mourning thereof. Such resolutions are most effective when achieved with support from significant others. The concept of positions implies that object relations are semi-stable^19^, i.e., dynamically reversible in response to changing internal or external conditions. Well-integrated individuals may experience splitting either as a consequence of transient prefrontal dysfunction under acute stress or as a functional defense mechanism in the context of chronic stress but normal prefrontal function. With respect to the former, acute severe stress may lead to increased catecholamine release in the PFC and thereby transiently impact prefrontal networks and working memory, while at the same time strengthening affective responses of the amygdala (Arnsten, 2015). Such neural “reflective-to-reflexive” switch may result in a transient state of regressive polarization of experience (“transient splitting”), but not necessarily permanently alter the neural organization of the PFC, as may happen with chronic severe and early stress (Czeh and Fuchs, 2016). In animal models, acute stress induces neural effects that may spontaneously correct given enough time for recovery (Czeh and Fuchs, 2016). With the resolution of acute stress, one would expect a relatively rapid restoration of prefrontal balancing of the mind. Under chronic severe stress, splitting may be created by covert yet teleological cognitive modulation of the weighted sensory-affective input into nonconscious working memory even in the context of intact PFC. Such splitting is a *bona fide* defense mechanism that polarizes reality into less complex and thus easier to manage dyads. In contrast, personality disorder is characterized by a relatively stable hypofrontality and splitting (Kernberg, 1975). However, phenotypic criteria for personality disorder tend to ebb and flow across the diagnostic threshold over time, although full health and social integration remain elusive (McGlashan et al., 2005; Temes and Zanarini, 2018). Such spontaneous symptomatic improvements and recurrences reflect alternations between compensated and decompensated functioning (rather than shifts between partial and integrated object relations), as discussed later. A dynamic, time- and context-sensitive nosology of personality disorder has been recently advocated (Svrakic and Divac-Jovanovic, 2019).

### Psychoanalytic Dual-Drive Theory Through the Prism of Cognitive Neuroscience and Unconditioned Affects

Reflecting the intrinsic organization of the encoding process, emotion-enhanced episodic memories show increased binding with their encoding context as well as inherent associations to other emotionally arousing and semantically (naturally) related memories (Talmi et al., 2019). Affect-enhanced memories attract attention obligatorily and are prioritized both for encoding into and retrieval in the face of a variety of contextual clues (Talmi et al., 2019). Semantic memory proper (i.e., knowledge of facts independent of context in which it was acquired) expands in a web-like pattern whereby neural representations of newly learned features are added to existing ones as successive stages of feature learning (Bauer and Just, 2015) and typically have no emotional biases for the information being learned. Semanticized self-related enhanced episodic memories expand in a similar web-like fashion, forming self-related associative knowledge structures or schemas, but preferentially assimilate information that is accordant with existing schema (Tse et al., 2011) while discounting contradictory experience (Disner et al., 2011). This implies an important role for affective input in schema-forming and updates, for both rewarding and aversive experiences (Phelps and LeDoux, 2005; Tonegawa et al., 2018). Based on Talmi et al. (2019), all naturally related and all affectively similar or similarly intense episodic memories tend to cross-promote each other by semantic (top-down) or by affective (bottom-up) stimuli, which may be objective or based on subjective inference (Sanders et al., 2020). The *en masse* activation may involve either real-time stimuli or may occur internally, by default mode processing which enables a person to time-travel through memories and their associated affects. This *en masse* effect on the web of schematized self- and object-representations and their associated affects by a single activating clue might be the mechanism of what Klein (1935, 1946) observed as a pervasive activation of a good or bad “internal object,” which of course also co-activates a pervasively good or bad experience of one's own self. It is plausible that Klein used the singular to illustrate the cross-activated plurality of destructive-aggressive vs. constructive-libidinal self- and object-representations, motivations, expectations, and action patterns internalized over time. Hartmann (1950) and Jacobson (1964) viewed libido and aggression as primary but not innate motivation systems. Rather, they argued that the two drives develop from heritable dispositions via interactions with others, pleasurable experiences consolidate into libido, unpleasurable into aggression. Following Kernberg's (2014) stratified concept of motivation systems the mutual cross-promotion of naturally related and affectively similar or similarly intense memories may be the mechanism by which unconditioned affects, considered by Panksepp (2011) to be the primary motivation system, coalesce as constituent components of supraordinate motivation systems of libido and aggression, the latter encompassing all constructive vs. destructive tendencies of a person.

## Cognitive Schema Theory and Object Relations Theory: Is There a Need For Both?

Object relations theory and cognitive schema theory are complementary in some and unique in other instances. Both theories concern schematized episodic experiences and self-reinforcing expansion patterns during infancy and toddlerhood (object relations) and childhood and adulthood (core-beliefs), both coalescing into cognitive-affective schemas for processing new information. As one of the main differences with object relations theory, Beck's cognitive theory considers core-beliefs, relatively advanced valuative concepts about self and others, to be the primordial source of self-reinforcing biases in interpreting new experiences (Beck, 2011; Disner et al., 2011). In contrast, object relations theory considers preconceptual survival- and safety- level experiences of infants and toddlers to be the primordial schema that regulates anticipation of safety vs. endangerment in subsequent experience. It appears that Beck's concept of core beliefs, frequently associated with explicit causative memories, falls short of recognizing preconceptual experiences in infants and toddlers that have been schematized but forgotten as specific memories. While complementing object relations theory in normal development, cognitive theory does not account for preconceptual, phase-sensitive aberrances in brain/mind development, such as partial object relations in personality disorder. On the other hand, object relations theory fails to recognize that maladaptive core-beliefs and schemas may develop despite integrated object relations, as in syndromes of depression, generalized anxiety, and panic disorder.

### Normal Development: Continuum From Self- and Object-Constancy to Adaptive (Functional) Core Beliefs and Schemas

Core-beliefs, for many the most innovative of Beck's concepts, are extracted from formative experiences during childhood (Beck, 2011) and formed into early concepts about self and others grouped into three main categories of self-sufficiency, lovability, and self-worth (Beck, 2011; Disner et al., 2011). The concept of cognitive schemas was deduced from clinical observations of depressed adults (Beck, 1964). According to theory, cognitive schemas are composed of sets of related core beliefs, and operate as stable, mainly latent templates for selective recognition of information that supports one's core beliefs, while discounting contradictions (Beck, 2011). Neuroscience of systems-consolidation of episodic memories has established that neocortical schematic frameworks extracted from persistent episodic events continue to receive input from the amygdala that provides contextual affects evoked by these events (Kitamura et al., 2017). The term “cognitive schema” in Beck's theory is thus somewhat misleading, as all schematized episodic experience is associated with respective affect information. Cognitive theorists talk about “*depressive*^20^ schemas… created by adversity^21^… characterized by negative self-referential beliefs” (Disner et al., 2011, p. 467), implying that affective valence is an integral constituent of core beliefs and cognitive schemas, much like in object relations. Schema forming and self-reinforcing expansion patterns have been demonstrated for rewarding and aversive episodic experiences, as discussed.

In contrast to preconceptual object relations, core beliefs emerge at more advanced stages of cognitive development, require self-referential cognition, and ability to form complex causal concepts. Based on modern neuroscience of schema-forming and schema-expansion in animals and humans (van Kesteren et al., 2010, 2012, 2016; Tse et al., 2011; Wang et al., 2012; van Kesteren and Meeter, 2020), under normal circumstances, schematized self- and object-constancy (the earliest self-related concepts extracted from integrated object relations) regulate own expansion into cognitively advanced positive self-valuations reflecting themes of self-sufficiency, lovability, and self-worth (adaptive core beliefs), and finally generalize into higher-order adaptive schemas that preferentially assimilate positive while discounting negative information. Each phase in such cognitive development likely involves processing in mPFC (Tse et al., 2011; Tonegawa et al., 2018), which seems to be the main hub for generalizing self-related knowledge based on past experience. Affect-memory is provided by the amygdala input to mPFC (Kitamura et al., 2017) and from here into working memory (Smith et al., 2018). Given the self-reinforcing patterns of schema updates, core beliefs are strongly influenced, but not predetermined^22^ by the antecedent implicit schema of object relations.

A developmental transition between finalization of object relations and emergence of core beliefs occurs between 2 and 4 years of age. During this period, the attainment of self- and object-constancy is paralleled by capacity for self-referenced cognition, self-reflection, introspection, and abstract appraisals of self and others (Tahka, 1988; Disner et al., 2011). These valuations can be adaptive, i.e., with a positive affective valence (“I am worthy,” “People are helpful”) likely reflecting the continuum from self-constancy to positive self-appraisals in the context of good-enough parenting and/or constitutionally tempered affects. Core-beliefs with negative affective valence are maladaptive or dysfunctional (e.g., “I am incompetent,” “I am unlikable”). Maladaptive core beliefs do not necessarily reflect pathology of object relations (Box 6).

Box 6 **CBT in mood and anxiety syndromes and personality disorder.**

- CBT targets Beck's cognitive hierarchy of schematized experiences (automatic thoughts, core- and intermediate beliefs, higher order schemas) (Beck, 2011; Disner et al., 2011) as units of intervention. Evaluative “automatic thoughts” that arise in *specific* situations^3^ (e.g., facing a new assignment at work), not a result of reasoning and usually only phenomenally conscious, are associated with shifts in emotions, motivation, physiological reactions, and behavior (Beck, 2011). Analysis of aroused emotions in specific situations may uncover automatic thoughts, maladaptive core beliefs, and higher-order schemas that underlie depression and anxiety syndromes. Despite negative self-appraisal, depressed or anxious individuals usually have a coherent identity, moral values, empathy, theory of mind, adaptive character traits, and prosocial behaviors. Maladaptive core beliefs may have been formed and superposed upon integrated object relations, perhaps in children who for any reason have not worked through the anxieties of depressive position into self- and object-constancy. These children experience envy, guilt, ambivalence, aggression, and fear of losing important objects. In older children and adults, social adversity or trauma, such as sibling rivalry, school bullying, adult abuse, singly or in concert with genetic dispositions to anxiety and depression, may cause regressive undoing of self-constancy and increase the likelihood of maladaptive core beliefs and schemas in social or interpersonal situations. Such children/adults are susceptible to negative self-appraisals, usually around themes of unlovability, worthlessness, or vulnerability to harm. CBT has proven effective (Dugas and Robichaud, 2007; Beck, 2011) and, with some disorder-specific modifications, is treatment of choice (Stewart and Chambless, 2009) in *bona fide* depression and anxiety syndromes (i.e., with no underlying partial object relations as in personality disorder). Once maladaptive beliefs and schemas that perpetuate depression and anxiety are “unlearned” through CBT exercises, self- and object-constancy may be restored and more functional core beliefs and schemas developed (Beck, 2011). A metanalysis of CBT in Major Depression shows a relapse-lowering effect superior to antidepressants; the effect continues even after acute CBT was discontinued (Leichsenring and Leibing, 2003), much like psychodynamic therapy continues to benefit patients with personality disorder after therapy completion (Shedler, 2010).
- Meta-analyses of CBT vs. psychodynamic therapy for personality disorder show mixed results, from comparable short-term effectiveness (Leichsenring and Leibing, 2003) to longer-term CBT inferiority (Shedler, 2010, Table 1). Maladaptive attitudes in personality disorder are epiphenomena, molded by fantasized compensatory self-image, not real experience. Once subtype-specific attitudes (arrogance, entitlement, theatricality, and antisociality) are corrected by CBT (real-time effectiveness), there still remain underlying survival- and safety-level anxieties that prioritize the relevance of one's own fragility and real or perceived rejection in comparison to which all other experiences are of secondary importance. Such continuing influence may reactivate the need for compensation, as the correction of maladaptive attitudes leaves the individual defenseless against the deeply rooted sense of endangerment and rejection. Thus, unmodified CBT is a symptom-based therapy of personality disorder, as it treats epiphenomena rather than the preconceptual core of the syndrome.
- To improve CBT for personality disorder, Beck himself (Beck et al., 2004) proposed a tripartite approach, combining rational analysis, analysis of development *via* emotional recollections, and examination of beliefs within a warm relationship with a therapist. Young et al. (Young et al., 2003) raised this proposal to another level by developing Schema Focused Therapy (SFT) that *combines* CBT and psychodynamic approaches, but also draws from Gestalt and attachment theories, humanistic approaches, and emotion-focused therapy. SFT expands standard CBT by emphasis on early emotional experiences, real or perceived inadequate parenting, and relies on the healing influence of the therapeutic relationship especially with respect to core needs for safety, nurturance, and protection (Young et al., 2003). SFT is tailored to the development of secure attachment to the therapist (suggestive of achieved integration of object relations) while at the same time correcting dysfunctional attitudes *via* standard CBT techniques. Such trans-theoretical multifocal approaches (Prochaska and Norcross, 2009) may help explain the real-time and long-term efficacy of SFT in personality disorder, with benefits persisting after therapy completion in a self-perpetuating manner (Giesen-Bloo et al., 2006). The author's claim that SFT is superior to transference-focused therapy of personality disorder has been challenged (Yeomans, 2007). Important here is that SFT, the most “psychodynamized” of CBTs, shows more efficacy than unmodified CBT in personality disorder.

Core-, intermediate beliefs,^23^ and cognitive schemas are generally considered to be implicit. However, core beliefs can reach phenomenal self-awareness, passively noticed (“felt to be true”), as “something that it is like for one to be in a particular mental state” (LeDoux and Brown, 2017), or in Beck's (1987) words, just the way things “are.” They are uncovered through CBT-methods (journaling of emotional reactions and automatic thoughts across related situations) or by careful self-introspection.

### The Special Case of Personality Disorder

Cognitive theory does not account for preconceptual traces of adversity in infants and toddlers. In personality disorder, chronic early adversity may lead to persisting dichotomization of self- and object-representations that coexist as unsystematized, predominantly negative fragments (Klein, 1935, 1946) reflecting themes of endangerment and rejection. The priority of coping with such urgency may interfere with the development of self- and object-constancy, a known deficit in personality disorder (Kernberg, 1975) and, down the time-line of complex self-referential appraisals (core beliefs) (Box 6). These deficits contribute to life-long identity diffusion typical of individuals with personality disorder. In the context of an ill-defined, fragmented sense of self and others, partial object relations are manifested as diffuse concerns about own physical and mental fragility, e.g., hypochondriasis, alternation between idealization and devaluation of same significant objects, disintegration anxiety, distrust, rejection sensitivity, among others (Svrakic and Divac-Jovanovic, 2019). The early distressed mind resorts to omnipotent fantasy to unilaterally fabricate a positive experience as a focal point for continuing mind development. This creates a compensatory self-image, specific for individual subtypes, e.g., narcissistic, schizoid, paranoid, histrionic, among others (Svrakic and Divac-Jovanovic, 2019). Maladaptive attitudes about self and others in personality disorder are epiphenomena. They may resemble but are qualitatively different from core beliefs, as they are molded by a fantasized, compensatory self-image that organizes mental life and interpersonal relations, e.g., “I have to be special” or “I have to impress” in narcissistic and histrionic personalities, respectively (Beck et al., 2004). Novel or challenging situations activate these compensatory attitudes to protect the underlying anxieties of endangerment and rejection (Svrakic and Divac-Jovanovic, 2019) which remain split off and with no immediate impact on behavior.

The inability of cognitive theory to explain personality disorder was first recognized by Young (1990) who hypothesized that, rather than having “ordinary” maladaptive core beliefs, individuals with personality disorder have “early maladaptive schemas.”^24^ The latter are postulated to arise from unmet core needs for safety, nurturance, and affection, and operate as automatic, preconceptual, and non-conscious anticipation of rejection, emotional deprivation, mistrust, and victimization by others (Beck et al., 2004; Bernstein, 2005; Esmaeilian et al., 2019), much like partial object relations. Young et al. (2003) also describe non-conscious “early coping mechanisms” that protect against intense affects associated with early maladaptive schemas, much like the mentioned protective fantasy compensates for partial object relations in personality disorder. Young et al. (2003) compare the three coping mechanisms to the evolutionary survival mechanisms of “freeze, flight, or fight,” respectively, much like attachment pathology associated with partial object relations. Early maladaptive schemas and related schema coping mechanisms are considered, again much like partial object relations, to form the core of personality disorder (Bernstein, 2005). Schema coping mechanisms eventually become dominant traits and automatic patterns of adult behavior (Young et al., 2003), much like compensatory self-representations in object relations theory of personality disorder. Young's approach to personality disorder brought closer Beck's cognitive model and object relations theory but used different words for similar concepts.

## Personality Disorder: Developing the Mind With Partial Object Relations

In most cases of less-than-perfect parenting and/or heritably high dispositions to affect- reactivity, developmental imperfections are incorporated into the brain/mind which stay within the quantitative range of normalcy. This common biological phenomenon, known as “developmental homeostasis” (Alcock, 2001), is achieved by multidirectional homeostatic optimizations affecting all components of a complex self-organizing adaptive system (Bak, 1996; Svrakic et al., 1996; Chan, 2001). However, heritably excessive negative affect-reactivity, and/or bad-enough parenting, social adversity and/or psychological trauma, during phase-sensitive periods (Teicher and Samson, 2016), may overpower the brain's/mind's homeostatic ability to assimilate imperfections. This may result in brain and mind pathology of personality disorder, the former exemplified by a hypo-functional PFC and amygdala hyper-sensitivity to negative affects (Teicher and Samson, 2016), the latter by pathological persistence of dyadic self- and object-representations, which remain unsystematized and coexist as split off fragments in the mind (Svrakic and Divac-Jovanovic, 2019). As persistent episodic memories eventually are semanticized, it follows that uncontrollable stress, re-experienced across multiple interactions with bad-enough caregivers, is extracted into a gist-like neocortical schema (Tonegawa et al., 2018) with a net-valence of fear, here of an endangered but helpless and rejected self-surrounded by a distressing and unhelpful world of significant objects. The phenomenology of personality disorder suggests that the child searches for safety unilaterally, via non-conscious primitive defense mechanisms, most notably via omnipotent compensatory fantasy (Svrakic and Divac-Jovanovic, 2019). Childhood precursors of personality disorder, including an extreme, unrealistic sense of self, are observable as early as 6-years-of-age (Uytun and Öztop, 2015). Winnicott (1955) observed an omnipotent “false self” in children as a defensive organization developed to counter the threat of annihilation by early adversity. We first discuss non-conscious working memory where, we hypothesize, fantasy and other defense mechanisms operate, and then introduce the concept of homeostatic compensation as a mechanism underlying personality disorder.

### Non-conscious Working Memory: the Hidden Layer of the Mind

Non-conscious working memory is a largely uncharted territory with a potentially major role in the assembly of mental life. It has been referred to as the “foyer” of consciousness or the “back-bone” for high-level cognition, because the maintenance of non-consciously perceived information has been shown to engage the PFC (Bergstrom and Eriksson, 2014), the brain region instrumental for abstract cognition and conscious working memory (Wang et al., 2006). Evidence for non-conscious working memory is growing (reviewed in LeDoux and Brown, 2017). Using fMRI and transcranial magnetic stimulation (TMS, Rose et al., 2016) demonstrated how neural representation of an item in working memory drops to baseline and the item appears forgotten when attention shifts away. A targeted single pulse of TMS can produce a brief re-emergence of the unattended item in focal attention. This effect suggests that activity-silent mechanisms maintain non-conscious working memory or, in the author's words, that “information in working memory (outside of focal attention) can be maintained in a latent state via mechanisms other than sustained, elevated activity” (Rose et al., 2016, p. 1136). The re-activation effect was found to occur for items participants were informed would be relevant later in the trial, suggesting that non-conscious representation is dynamic and susceptible to teleological cognitive influences. Bergstrom and Eriksson (2018) also found evidence for “cognitive control processes during non-conscious memory recognition” (p. 3225). Artificial neural networks utilized in machine learning contain a hidden layer located between the input and output layers. The hidden layer performs computations on weighted inputs (synaptic weights in biological networks of neurons) and produces net-input based on non-linear transformations. The net-input is applied with activation functions to produce the output (now input for the output layer) that is more or less fundamentally different from the original input (“disjoint” and “convex” decisions, respectively) (Huang et al., 2000). Such hidden layer (or layers)^25^ that appear to exist in biological networks of neurons (Rose et al., 2016; Bergstrom and Eriksson, 2018; Gonzales et al., 2018), might host the operations of non-conscious working memory, most notably the homeostatic optimization processes, the driving force behind self-organization of complex dynamical systems in biology and physics (Bak, 1996), including human personality (Svrakic et al., 1996). One example of such homeostatic optimization may be the concept of defense mechanisms as defined by psychoanalysis. Covert but teleological cognitive influences on information processing in non-conscious working memory have been documented, as discussed. Such influence on input information might be the mechanism of non-conscious distortions of reality, i.e., “defense mechanisms,” that guard against conscious representation of inner conflicts or intolerable reality by redefining the input (mature defenses), repressing it (neurotic defenses), or polarizing it into less complex and thus easier to manage dyads (splitting and other immature defenses) (Svrakic and Divac-Jovanovic, 2019). Defense mechanisms have been historically regarded as one of the non-conscious functions of the Ego, otherwise considered to be largely conscious by psychoanalysis. Freud drew from the principle of homeostasis to formulate the concept of Ego as a “structure” within the mind, rather than a general tendency, inherent to all self-organizing complex adaptive systems in biology, toward a relatively stable equilibrium between interdependent elements; this makes Ego a non-conscious function of the mind as a whole^26^.

### Neuropathology of Personality Disorder

Infants and children exposed to chronic severe stress may develop phase sensitive, widespread, and enduring neuropathological effects on vulnerable brain regions, notably the hippocampus (Luby et al., 2013), and medial^27^ and dorsolateral prefrontal cortices (Teicher and Samson, 2016), coupled with a premature engagement of the amygdala in mental processes (Tottenham, 2012), enhanced amygdala responsivity to emotional stimuli, and diminished striatal response to anticipated rewards (Teicher and Samson, 2016). Prefrontal dysfunction (“hypofrontality”) is manifested as a suboptimal top-down control of affects, deficits in working memory, executive function, decreased capacity for mentalization and theory of mind of others, lack of empathy and other prosocial emotions (Preißler et al., 2010; Dimaggio et al., 2012; Marini et al., 2016). Subcortical dysfunction is manifested as amygdala-mediated hyper-sensitivity to negative emotions, especially to signs of rejection, and lack of genuine interest in commonly rewarding activities (Teicher and Samson, 2016), such as parenting or work, among others. A strong amygdala response in infancy, in the context of an immature mPFC and its yet-to-develop inhibitory inputs on the amygdala affects, has been shown to be mediated by caregivers (mother), either accentuating affective responses (bad-enough caregivers) or alleviating them (good-enough caregivers) (Tottenham, 2012). In other words, a good-enough parent assumes the role of mPFC while the latter is still not developed sufficiently. A full maturation of amygdala-mPFC connectivity may take two decades of interactive development, when amygdala hyper-reactivity is top-down regulated by mPFC (reviewed in Tottenham, 2012). Figuratively, the developing amygdala is “the student of the world and a teacher of the cortex” (Tottenham and Gabard-Durnam, 2017). The mPFC-amygdala circuitry is commonly affected by early life stress (Tottenham, 2020). Amygdala is rich in stress-hormone receptors that make it very susceptible to stress especially in neonates (Tottenham, 2012). In individuals with personality disorder, the mPFC does not mature but continues to be “tutored” by the amygdala. In some cases, chronic stress occurring after a good-enough childhood may damage established functional connectivity by increased olygodendrogenesis and reduction in neurogenesis in the hippocampus, a super-hub in many functional networks (Chetty et al., 2014). Such “fracturing” of functional connectivity may explain cases of personality disorder arising later in development.

### Homeostatic Compensation Through Fantasy as the Potential Mechanism of Personality Disorder

Personality disorder may result from a homeostatic attempt of the early brain/mind to compensate for the predominance of negative self- and object-representations associated with survival- and safety-level anxieties (Svrakic and Divac-Jovanovic, 2019), an adaptive response to facilitate survival and reproduction in the face of adversity (Teicher and Samson, 2016), the latter frequently perpetrated by caregivers. The lack of an available caregiver to provide comfort may be a critical to make these experiences overwhelming or traumatic (Lane et al., 2015). In such a context, the sense of safety must be created by compensatory mechanisms, a common strategy in brain and mental functioning (Fassbender and Schweitzer, 2006). Due to stress-induced early prefrontal dysfunction, suppression of distressing memories, a healthy prefrontal mechanism mediating resilience after trauma (Mary et al., 2020), is ineffective. Instead, the drop in fitness may drive the distressed mind toward omnipotent fantasy (Svrakic and Divac-Jovanovic, 2019) and other survival-type adaptations, such as preference for interpersonal distance (Maier et al., 2020)^28^. Such homeostatic maneuvering staves off the risk of disintegration but shunts the developing mind into the deviance of unrealistic self-image and maladaptive expectations of support for this image in interpersonal relationships. Together, such intra- and interpersonal dynamics create the characteristic phenomenology and symptoms of personality disorder (Svrakic and Divac-Jovanovic, 2019)^29^. The wishful imagery and revengeful affects of omnipotent fantasy unilaterally fabricate the sense of control over malevolent objects and adversity. The process can be conceived as unilateral conditioning of fear inhibition by fantasized safety, akin to “learned safety,” a protective learning process in animals exposed to adversity to respond to signals of safety that can help prevent and reverse chronic stress (Pollak et al., 2008). Fantasy is driven by homeostatic optimization processes to restore the equilibrium within the mind at risk of disintegration rather than by real experience. As a defensive mechanism most likely operating in non-conscious working memory, above the level of neural encoding of real experience, fantasy can compensate for negative representations but cannot transform them into actual positive self-and object-images. Learned safety in rodents has been shown to promote neurogenesis in the hippocampus and to modulate monoaminergic genes in the basolateral amygdala (Pollak et al., 2008). With this in mind, it plausible that variant-specific, fantasized compensatory self-images in humans with personality disorder also have a neural substrate, e.g., as schematized variant-specific behaviors and emotions (narcissistic, histrionic, etc.) across situations, potentially explaining the relative stability of such images over time.

Based on research in animals (Tse et al., 2011) and humans (van Kesteren and Meeter, 2020), the inaugural schema-affect-encodings of early adversity are likely to thereafter implicitly promote preferential assimilation of perceived rejection, while positive experiences are assimilated as exceptions. As such, partial object relations interfere with normal self-organization of functional connectivity networks that underlie mental faculties, rendering thought, emotion, and motivation dichotomized, alternating between *decompensated* (e.g., anxieties about one's own ability and safety, paranoid interpretation of the environment, aggressive-destructive tendencies toward significant objects) and *compensated* (when fantasized self-imagery is sufficient to organize the mind while awareness of own vulnerability to rejection is only vaguely recognized) (Svrakic and Divac-Jovanovic, 2019). The shifts between regressive and compensated functioning usually occur in high- vs. low-stress situations and unstructured (destabilizing) and structured (stabilizing) environments, respectively (Svrakic and Divac-Jovanovic, 2019).

## Neuroscience of Psychodynamic Psychotherapy

All individual variants of personality disorder share the same core deficit of partial object relations, alternatively called “borderline personality organization” (Kernberg, 1975) or “fragmented personality” (Svrakic and Divac-Jovanovic, 2019), while differing with respect to the compensatory self-image, e.g., narcissistic, schizoid, paranoid, among other subtypes. Personality disorder is, thus, considered a deficit-type psychopathology because the dichotomized mind is attempting to amalgamate itself against disintegration^30^. In search of an effective approach to restructure implicit partial object relations, a number of psychotherapies have been adapted for personality disorder, but in retrospect, the psychodynamic framework remains a key underlying approach (Shedler, 2010; Magnavita, 2012). When transcripts and video recordings of session are analyzed, regardless of the therapy the therapist believed was being administered, the therapist's adherence to the psychodynamic prototype predicts successful outcome in both psychodynamic and CBT approaches to personality disorder (Shedler, 2010).

### Clinical Data

Meta-analyses of controlled studies of long-term psychodynamic psychotherapy of personality disorder demonstrate substantial improvements in general mental functioning and discrete symptoms, such as impulsivity and interpersonal functioning (Leichsenring and Rabung, 2008; Magnavita, 2012). Psychodynamic therapy has been associated with sustained maturation of primitive defense mechanisms (Bond and Perry, 2004), one of the three hallmarks of partial object relations^31^. Other studies (Levy et al., 2006) show that transference focused psychodynamic psychotherapy, but not dialectic behavior therapy or modified psychodynamic support, is associated with significant transitions to secure attachment and improved reflective function. The cited results are suggestive of cognitive integration of partial object relations, likely mediated by improved organization of mPFC and its top-down regulation of emotion, as supported by neuroimaging (Lai et al., 2007; Perez et al., 2016). Brain fMRIs of individuals with borderline personality after transference focused therapy showed a positive correlation between improvements in affective lability and left posterior-medial orbitofrontal cortex/ventral striatum activation, and a negative correlation with right amygdala/parahippocampal activation (Perez et al., 2016). The benefits of long-term psychodynamic therapy not only continue after therapy is completed but also tend to increase over time in a self-perpetuating fashion (Shedler, 2010). In contrast, the benefits of non-psychodynamic but real-time effective therapies, such as CBT, tend to dissipate over time (Shedler, 2010, Box 6).

### Potential Neural Mechanisms of Psychodynamic Psychotherapy

Advancing cognitive- and systems-neuroscience of episodic memory has identified patterns of information processing in cortical (schematic) and subcortical (emotional) learning and memory systems that dynamically interact to mediate widespread neural and mental adaptations to changing environments. Independently acquired units of knowledge on this subject may help outline a plausible scenario for neural mechanisms of psychodynamic psychotherapy. Some of these findings have already been discussed, e.g., encoding and semanticization of enhanced episodic memories, schema-expansion patterns (Box 3), some are discussed below.

- Central to our discussion is the dynamic and reversible plasticity of the hippocampal-amygdala-mPFC circuitry involved in encoding and systems-consolidation of affect-enhanced memories (Tonegawa et al., 2018). Distinct groups of amygdala neurons are genetically programmed to encode either fear or rewarding affects (Kim et al., 2016). These groups are anti-correlated (processing reward supresses processing in fear-dedicated neurons and *vice versa* (Kim et al., 2016) and fixed in dedication (i.e., cannot be re-trained to engramize affects not in accord with their program, Redondo et al., 2014). In contrast, hippocampal engrams that encode a semantic-like content (e.g., “what” – an aversive event), context (e.g., home cage), and drive avoidant behaviors, can be re-trained to switch and engramize a positive-valence content-context (e.g., rewarding female presence, home cage) and drive home-cage preference (Redondo et al., 2014). The switched hippocampal processing engages amygdala partners that encode the corresponding affect-memory (now reward), while in parallel weakening original connections with negative affect-memory (Redondo et al., 2014). The mPFC, informed by inputs from the hippocampus and amygdala, (i) system-consolidates these new positive episodic memories (Kitamura et al., 2017) and (ii) is believed to form a schema-affect template hat thereafter directs attentional priorities to Gunseli and Aly (2020), regulates relevance, and guides preferential assimilation of positive experiences (Tse et al., 2011) while discounting contradictions, as discussed. The neural substrate of such dynamic system-level adaptive flexibility of memory-based implicit schemas is unknown. It likely involves, among others, immediate early genes (Tse et al., 2011; Tonegawa et al., 2018), epigenetic modifications (Tonegawa et al., 2018), mPFC neurons phase-locking to hippocampal theta-rhytms (Siapas et al., 2005), and sharp-wave ripple-mediated replay of activity of hippocampal engrams occurring in temporal proximity with cortical delta activity during slow-wave sleep (Çalişkan and Stork, 2018).
- Functionally distinct neuron ensembles within a single memory engram in the hippocampus promote adaptive generalization or discrimination of that memory in changing environments (Sun et al., 2020). Such functional heterogeneity within a single engram is thought to represent a “higher order” memory trace within the “essential memory trace,” the former enabling adaptive regulation of memory-guided behavior in environments that are accordant or discordant with the memory (Sun et al., 2020). In rodents, neurons genetically defined by Fos-dependent transcription and receiving excitatory cortical synaptic input, promote memory generalization in the context of accordant new experiences. In contrast, other neurons within the same engram but genetically defined by Npas-4^32^ and receiving inhibitory synaptic inputs from local interneurons, mediate discrimination of the same memory in the context of discordant new experiences (Sun et al., 2020).
- Remapping of hippocampal engrams that encode context and content reflects subjective beliefs about the hidden states of environment (“hidden state inference”), rather than objective, observable properties of the environment (Sanders et al., 2020). Hidden states are inferred from observations that are based on and thus also biased by prior experience (safety, danger). The regularity of a hidden state can be learned over the course of experience, leading to increased certainty about hidden state assignments (Sanders et al., 2020).
- Cortically schematized enhanced episodic memories are strongly associated with respective amygdala affect-memories (schema-affect complexes) (Kitamura et al., 2017). Evidence from cognitive neuroscience (Talmi et al., 2019) indicates that emotion-enhanced episodic memories can be activated by affective clues (bottom-up activation) perhaps involving the sensory cortices-thalamus-amygdala “short-cut” (Phelps and LeDoux, 2005) as well as by natural relatedness (semantic clues) likely involving the sensory cortices-thalamus-mPFC-amygdala pathway (Kitamura et al., 2017). Primordial schema-affect units of object relations are non-declarative, i.e., not accessible by attention and conscious working memory. The thalamus-amygdala short-cut allows sensory stimuli to activate affects in parallel or before the signal arrives to the cortex, and may be important for precognitive emotional processing (LeDoux, 1992) that in turn may provide implicit access to the implicit schema-affect units of early object relations.

It is unknown but plausible that the above cortical and subcortical mechanisms work in concert, i.e., that they dynamically interact to achieve multidirectional cognitive-affective reconfigurations of the brain and mind as a flexible adaptive response to persistent new episodic experience.

#### Psychodynamic Interventions

The main “tools” of psychodynamic psychotherapy are transference interpretation and corrective emotional experience (CEE). Transference^33^ interpretation is used to uncover implicit anticipation of rejection as it is directed toward the therapist in the here-and-now or, alternatively, to uncover repetitions of conflictual past relationships in the here-and-now (Auchincloss and Samberg, 2012). In either case, transference interpretation may lead to insights, considered to be fundamental to the therapeutic effect. Insights are defined as sudden, catharsis-like intuitive apprehending of inner nature of things that makes non-conscious mental life accessible to conscious understanding (Auchincloss and Samberg, 2012). The therapeutic mechanism of insights is unknown. It is generally believed that individuals with personality disorder non-consciously activate partial object relations (that is anticipation of endangerment and rejection) in transference. One possibility is that such catharsis-like (emotional) understanding of inner nature of how one's mind operates at its core may destabilize and implicitly update (restructure) the forgotten emotional memories of infancy and toddlerhood via amygdala-mPFC input (discussed below).

CEE (a concept of Alexander, 1950) refers to the healing aspect of psychodynamic therapy that works alongside interpretation and insight. CEE is a form of implicit emotional learning within the relationship with a significant object (the therapist) that could not have occurred outside the treatment setting (Christian et al., 2012)^34^. The relationship is characterized by the therapist's unconditional, empathic, and expert presence in the therapeutic process (figuratively, a good-enough third parent). The implicit anticipation of endangerment and rejection is activated within a safe, helpful, and empathic significant relationship. Once controversial, CEE has regained scientific interest (Christian et al., 2012). Its mechanism is still debated. One possibility is that learning of safety and acceptance within the therapeutic relationship occurs at a procedural or sub-symbolic level (Christian et al., 2012). As noted, the primordial negative schema-affects complex of partial object relations is automatically activated in transference. Such activation, albeit non-conscious, might make the implicit schema destabilized and open to updates (Lee et al., 2017). The mentioned thalamus-amygdala short-cut may be pivotal for such updates. Amygdala engages mPFC (anterior cingulate in particular) during associative learning (Keefer and Petrovich, 2017). In primates, an overall increased theta power and a directional phase locking develops between amygdala spikes and anterior cingulate input (local field potentials that are locked to theta band) and declines once aversive memory is formed (Taub et al., 2018). This unidirectional synchronization could be a plausible mechanism to transform CEE into an implicit amygdala input to mPFC, and update (reconstruct) the implicit schema of endangerment and rejection, acquired decades earlier at the same sub-symbolic level and in a similar setting.

Another mechanism of CEE could involve metaplasticity, defined as activity-dependent and persistent regulation (“priming”) of the ability of synapses to induce subsequent plasticity, i.e., long-term potentiation or depression, figuratively “plasticity of synaptic plasticity.” Metaplasticity mediates adaptive changes in the interneuron network that controls principal neuron activity and generates behaviorally-relevant local field potentials in the hippocampus-amygdala-mPFC hubs at different phases of emotional learning (reviewed in Çalişkan and Stork, 2018). Changes in the interneuron network may induce metaplasticity by (i) generally altering the oscillation characteristics in the local circuit and their synchrony with the target regions, and (ii) by controlling engram formation and their participation in the local circuitry (Çalişkan and Stork, 2018). In therapy, implicit experience of safety and acceptance may prime the hippocampus-amygdala-mPFC memory system to preferentially process emotionally positive sub-symbolic experiences.

In humans and animals, research has implicated the hippocampus-amygdala-vmPFC network in both extinction and cognitive reappraisal of emotional stimuli (Phelps and LeDoux, 2005). As noted, the benefits of unmodified CBT in personality disorder tend to dissipate with time (Shedler, 2010). The same hippocampus-amygdala-mPFC network is believed to mediate benefits of psychodynamic therapy, but here benefits tend to continue and increase after therapy completion. It appears that transference interpretation and insights, coupled with implicit corrective emotional experience, both unique to psychodynamic therapy, make the difference between the two approaches to personality disorder.

#### Disruption of Reconsolidation: A Plausible Intervention-Mechanism Scenario of Psychodynamic Therapy

Early in therapy, the existing implicit schema of partial object relations, typically accentuates the relevance of and selectively prepares attention toward rejection and distrust (Tse et al., 2011; Gunseli and Aly, 2020). Positive experiences are assimilated as exceptions and may be even discriminated against (Sun et al., 2020). Each time these negative schema-affect complexes are activated in therapy, the overlaps (i.e., perceived rejection) re-consolidate the existing schema. Nevertheless, persistent positive episodes, explicit (e.g., transference interpretation and insights) and implicit (e.g., corrective emotional sense of safety and acceptance) are mediated by a significant object (the therapist) in the context of positive primary affects (care). The latter may be encoded via the mentioned thalamic-amygdala shortcut. Amygdala spiking associated with positive emotional experience may phase-couple with mPFC theta oscillations and become an emotional implicit input (Taub et al., 2018). Additionally, persistent environment of safety and acceptance may prime the hippocampus-amygdala-mPFC memory system to selectively process emotionally positive experiences via metaplasticity (Çalişkan and Stork, 2018). Such implicit emotional learning may not only disrupt chronic reconsolidation of endangerment and rejection, but also may re-construct (update) the net-negative schema of partial object relations toward a net-positive valence (van Kesteren and Meeter, 2020). As mPFC is a key hub in functional networks that underlie mental faculties, such selectivity toward safety and acceptance may become a stable attractor for the maturation of these networks “from within,” much like partial object relations interfered with normal brain/mind development during phase-sensitive periods of infancy and toddlerhood. The process of change likely follows Bayesian dynamics, i.e., the more sub-symbolic evidence for safety and acceptance – the higher the probability of emotional learning and cognitive network maturation. It follows that many repetitions of implicit experience of safety and acceptance are needed for CEE to occur. In other words, integration of object relations in psychodynamic therapy is not new learning inhibiting but not eliminating old learning (as appears to be in CBT-mediated extinction) (Phelps and LeDoux, 2005) but restructured old emotional learning; this may explain the post-therapy continuation of benefits in a self-perpetuating fashion (Shedler, 2010). Once established as predominant, integrated object relations tend to promote own expansion by preparatory attention toward and preferential assimilation of positive representations of self and others in all significant relationships. Such generalization is possible because the master schema of early object relations is not object-specific but experience-specific, as episodic memories of particular events have been forgotten. Negative experiences may be discriminated against as early as at the level of hippocampus-amygdala processing (Sun et al., 2020), and may lead to increased certainty of assignments about hidden state of the environment (Sanders et al., 2020), now expected to be positive.

## Conclusion and Future Directions

A key going forward concerns testable research directions that flow logically from our model and discussion to (a) enhance mental health, (b) prevent, and (c) improve treatment of many forms of mental disorder, personality pathology in particular. Some directions for research include:

- determining how to maximize healthy plasticity of critical neurocircuits to enhance well-being perhaps via neurogenesis [e.g., diet, exercise (Hueston et al., 2017; Liu and Nusslock, 2018), mindfulness (Hölzel et al., 2011), other approaches to stress reduction];
- determining how to augment psychodynamic psychotherapy by co-treatments that enhance plasticity [(e.g., the mentioned stress-reduction strategies, psychedelics combined with psychotherapy (Svrakic et al., 2019); other pharmacological treatments targeted to mechanisms underlying synaptic plasticity, including positive modulators of NMDA receptors, or neurostimulation, particularly trancranial magnetic stimulation targeting neural circuits underlying attention and memory (Marshall et al., 2006; Bourzac, 2016)];
- determining how to integrate other modifying influences to enhance mental health or ameliorate psychopathology, notably hormonal [(e.g., oxytocin (Heinrichs and Domes, 2008)] and epigenetic approaches [e.g., enriched environments (Veena et al., 2008)];
- determining how to promote transgenerational transmission of epigenetic modifications produced by psychotherapy, which opens up new perspectives for prevention science (Jimenez et al., 2018);
- determining the optimal ratio of transtheoretical psychotherapy methods to promote integration of partial object relations (the main goal) while at the same time addressing the remaining compensatory phenomenology and behaviors (integrated object relations are expected to reduce the need for such compensation, but some pathological habits may persist as hardwired schemas, as discussed). Based on available evidence, such multifocal approach (Prochaska and Norcross, 2009) should, at the minimum, include CBT, psychodynamic, relational, interpersonal and experiential methods. Manualization of such therapy would standardize treatments across therapists and facilitate research;
- determining whether we can take advantage of enhanced plasticity in the developing human brain for preventive purposes. Work in infants and toddlers is already identifying early predictors of individuals at risk for psychopathology that may inform early intervention (Luby et al., 2020). Interventions focused on promoting parent-child interactions and cognitive/emotional development can potentially use developmental plasticity in a specific fashion to dampen, if not prevent defects that underlie personality disorder and other forms of psychopathology (Luby et al., 2020). Some prophylactic interventions could be implemented by informed parents and public policy as well, a bench-to-bedside expanded to home-and-public guidelines for childrearing. Such evidence-based guidelines are surprisingly rare and much is left to the belief systems of parents, frequently reflecting the parents' childhood experience as to how their own affectively-charged situations were handled. Some inconsistencies are as basic as whether or not to let the infants cry themselves to sleep. A wrong strategy here may potentially have life-changing mental outcomes for children. Early life intervention also offers hope of preventing dysfunction associated with abuse, neglect and adversity that in many instances are-direct normal brain and mind trajectories toward personality disorder.

This research is of high priority because personality disorder, a relatively severe but preventable and reversible brain and mind dysfunction, is common (Lenzenweger et al., 2007) and associated with comorbidity with other mental disorders (which are usually treatment-resistant in this setting), and with a host of family, parenting, and social problems (dysfunctional families, child-abuse, distorted or atypical parenting, addictions, crime, etc.) (Pincus and Wiggins, 1990; Stepp et al., 2011; Juurlink et al., 2018). Thus, efforts to harness advancing neuroscience to enhance well-being as well as to correct the dysfunction outlined in this perspective can have major public health impact.

## Limitations

Contextual fear conditioning may have its limitations to fully represent episodic memory (Dunsmoor and Kroes, 2019). However, real world emotional experiences likely engage Pavlovian classical conditioning and episodic memory mechanisms (Dunsmoor and Kroes, 2019). Recombinant DNA and optogenetic studies demonstrate that contextual fear conditioning may share neurobiological systems involved in classical conditioning and episodic memory. The conditioned context (location in the cage) paired with unconditioned stimulus (foot shock) are initially encoded by the hippocampus, while the memory of fear is encoded by the amygdala which also drives avoidant behavior; the episode is system-consolidated in mPFC (Kitamura et al., 2017). Remote memory expressed by the mPFC engram was “conditioned-context specific, suggesting that it is episodic-like” (Kitamura et al., 2017, p. 5), a position of Eichenbaum (2017) as well. This research raises the possibility that contextual fear conditioning may be conceived as a simple form of episodic memory.

## Author Contributions

All authors contributed to the design, write-up, and content of the manuscript.

## Conflict of Interest

CZ is on Scientific Advisory Board of Sage Therapeutics. The remaining author declares that the research was conducted in the absence of any commercial or financial relationships that could be construed as a potential conflict of interest.

## Footnotes

^1^The term “subsystems” rather than intrapsychic “structures” are used in this work, as there are no *bona fide* structures in the mind, which is entirely a function.

^2^Fairbairn's “relational/structure” model positing that relational experience with caretakers modulates the structure of the mind, played a key role in the development of Bowlby's attachment theory.

^3^In a broader sense, a significant object is anyone or anything into whom/which one invests strong emotions: a romantic partner, work, an idea, religion, social activism, etc.

^4^Not to be confused with operant conditioning.

^5^Semantic-like valence of affect-enhanced episodic memory, not itself an affect but a factual information whether the event was rewarding or frustrating, is encoded by the hippocampus as a component of the context; the episode is systems-consolidated in mPFC (Kitamura et al., 2017). Optogenetic stimulation and contextual activation of remote episodic memory engram in mPFC activates respective amygdala-encoded affect memories and behavior (Kitamura et al., 2017) likely via extensive mPFC-amygdala connections (Reppucci and Petrovich, 2016). Hence, the phrase “schema-affect complex” is used.

^6^Defined as the implicit subjective awareness of initiating, executing, and controlling one's own volitional actions, usually referring to motor acts and their consequences.

^7^Convergence regions, e.g., prefrontal, parietal, and parahippocampal cortices, among others, are highly connected brain regions where concepts are formed from multimodal neural percepts, i.e., “where neural becomes mental” by cognitive synthesis (LeDoux, 2002).

^8^Introjection and internalization are overlapping concepts (Auchincloss and Samberg, 2012); here, the term introjection is used consistently to avoid confusion.

^9^The mechanism is sometimes called “introjective identification”.

^10^Engrams or “memory traces” refer to a small group of neurons co-wired in a Hebbian fashion, showing lasting cellular changes as a consequence of activation of that subpopulation of neurons by episodic stimuli; reactivation of engram cells by a part of the original stimuli results in memory recall (Tonegawa et al., 2018).

^11^The hippocampus appears to encode the contextual semantic-like affective valence (factual knowledge that an event was aversive of rewarding) perhaps based on the input from the amygdala affect-memory via stratum oriens (Muller et al., 2018).

^12^Both the creation of silent cortical engrams and their maturation require an intact hippocampus and include, among other mechanisms, immediate early genes, epigenetic modifications, and a repeated sharp-wave ripple-mediated replay of activity of hippocampal engrams (Tonegawa et al., 2018).

^13^Co-existence of symbiosis (Mahler et al., 1975) and partial object relations (Klein, 1946) during the first 6 months-of-life has been challenged as incompatible, as object-representations are necessarily encoded as dyadic from birth, due to hardwired affective dichotomization of experience in the amygdala (Kitamura et al., 2017).

^14^An approximation, based on Mahler's (Mahler et al., 1975) separation-individuation timeline, likely varies in individual instances.

^15^Winnicott (1971) coined the phrase “good-enough” to emphasize the importance of natural instincts of normal devoted parents and stable, healthy families, in an attempt to counterbalance the potential idealization of parenting implicit in Klein's concepts of “good mother”. Occasional manageable frustrations are necessary for the child to develop self-reliance (Kohut, 1971). The phrase “bad enough” refers to neglectful or abusive caregivers and dysfunctional families, sufficiently stressogenic to cause structural and functional brain/mind pathology, as in personality disorder.

^16^In infants, rudimentary sense of ownership of instincts and affects might be available, perhaps mediated by their respective interoceptive correlates and affect-memory traces (Panksepp, 2011; Damasio and Carvalho, 2013).

^17^The postulate applies to normal development, but not to all psychopathology, as discussed with CBT.

^18^As such, survival- and safety-level object relations can be conceived as elementary units of the mind, not divisible to lower-order mental experience only to its distributed neural components (Svrakic and Divac-Jovanovic, 2019).

^19^A semi-stable equilibrium of a complex system is one with multiple, but not unlimited solutions to semi-stable “attractors” (here changing internal and/or external conditions), which gives the system some, but not unlimited adaptive flexibility.

^20^Our italics

^21^Adversity and trauma are used interchangeably for emotionally painful abuse or mistreatment.

^22^Due to positive and negative intervening factors, environmental and genetic (Beck, 2011; Kernberg, 2014).

^23^Intermediate beliefs, defined as cognitively unarticulated attitudes, rules, and assumptions that a person follows as a general approach to novel or challenging situations, correspond to the concept of scripts in humanistic therapies, i.e., life-rules (or traps) that develop between 2 and 5 years of age and forward (Berne, 1972).

^24^Young et al. (2003) use the phrase “early maladaptive” to contrast preconceptual unarticulated schemas that correspond to partial object relations with “ordinary” cognitively articulated maladaptive schemas of self and others, as in depression and anxiety syndromes.

^25^One hidden layer keeps the output with some contact with reality (“convex decisions”), much like in defense mechanisms, which is in biological networks preferred over being disjointed from reality (Huang et al., 2000) (bizarre outputs) as in psychosis (here, perhaps 2 hidden layers operate due to widespread aberrancies in brain organization).

^26^Conversely, the Id is increasingly viewed as being conscious (Solms, 2013).

^27^The pivotal role of mPFC in integrated object relations is implicated by studies of adults with personality disorder in whom structural and functional abnormalities of mPFC, among other neuropathology, are reported (Krause-Utz et al., 2014; Salvador et al., 2016; Teicher and Samson, 2016).

^28^This refers to emotional but not physical interpersonal distance, as others are vitally needed, frequently demanded, to validate the unrealistic self-image of the patient, hence the frequent interpersonal conflicts (Svrakic and Divac-Jovanovic, 2019).

^29^Despite suggestions for a possible link between specific traumas in childhood and the risk of specific phenomenology of personality disorder, e.g., narcissistic, schizoid, etc. (Hankin and Abela, 2005) such causal relationship has not been established.

^30^In contrast to the surplus-type psychopathology of neuroses, by which the mind defends itself against the surplus of anxiety.

^31^The other two are identity diffusion and subtle impairments in reality testing (Kernberg, 1975).

^32^*N-pas4*, an immediate early gene encoding a transcription factor Neuronal PAS domain-containing protein 4 that controls inhibitory synapses.

^33^Transference refers to implicit ways of relating to significant objects, or implicit anticipations and automatized behaviors derived from early object relations (Auchincloss and Samberg, 2012). Transference is regularly enacted with selective significant objects in adulthood, including the therapist, who is drawn into playing a role that reflects the patient's internal world (Gabbard, 2020). Countertransference refers to the therapist's conscious and nonconscious reactions to the patient's transference (Auchincloss and Samberg, 2012) and is therefore jointly created by both parties (Gabbard, 2020). Counter-transference analysis is also a useful source of information, either in terms of understanding the patient's nonconscious world or for insight into how the patient generally affects other people.

^34^A rare exception may be a mature romantic partner with unconditional love, who receives, processes, and responds to primitive defense mechanisms in a therapeutic fashion (partner as intuitive therapist).

^1^The crosstalk appears to be mediated by mPFC neurons being phase locked to the hippocampal theta-rhythm (4–10 Hz) that characterizes hippocampal activity during active exploratory behavior and REM sleep (van Kesteren et al., 2016).

^2^The subsystem of identity (“who am I inside”) aligns past and present self-images and personal goals into a coherent narrative of the self and thus enables prospection. This usually requires some falsification of autobiographical memories, suggesting that identity may be a function of the working self, which controls accessibility to memory to an extent, rather than an independent subsystem within the mind.

^3^In contrast to intermediate beliefs which are active as *general* rules, attitudes, and assumptions, across situations and relationships, as noted.
